# Metabolic profiling of pregnancy: cross-sectional and longitudinal evidence

**DOI:** 10.1186/s12916-016-0733-0

**Published:** 2016-12-13

**Authors:** Qin Wang, Peter Würtz, Kirsi Auro, Ville-Petteri Mäkinen, Antti J. Kangas, Pasi Soininen, Mika Tiainen, Tuulia Tynkkynen, Jari Jokelainen, Kristiina Santalahti, Marko Salmi, Stefan Blankenberg, Tanja Zeller, Jorma Viikari, Mika Kähönen, Terho Lehtimäki, Veikko Salomaa, Markus Perola, Sirpa Jalkanen, Marjo-Riitta Järvelin, Olli T. Raitakari, Johannes Kettunen, Debbie A. Lawlor, Mika Ala-Korpela

**Affiliations:** 1 Computational Medicine, Faculty of Medicine, University of Oulu and Biocenter Oulu, Oulu, Finland; 2 NMR Metabolomics Laboratory, School of Pharmacy, University of Eastern Finland, Kuopio, Finland; 3 National Institute for Health and Welfare, Helsinki, Finland; 4 Institute for Molecular Medicine (FIMM), University of Helsinki, Helsinki, Finland; 5 Department of Obstetrics and Gynecology, Helsinki University Central Hospital and University of Helsinki, Helsinki, Finland; 6 Heart Health Theme, South Australian Health and Medical Research Institute, Adelaide, Australia; 7 School of Biological Sciences, University of Adelaide, Adelaide, Australia; 8 Center for Life Course Health Research and Biocenter Oulu, University of Oulu, Oulu, Finland; 9 Unit of Primary Care, Oulu University Hospital, Oulu, Finland; 10 Department of Medical Microbiology and Immunology, and MediCity Research Laboratory, University of Turku, Turku, Finland; 11 Clinic for General and Interventional Cardiology, University Heart Center Hamburg, Hamburg, Germany; 12 German Center for Cardiovascular Research (DZHK e.V.), partner site Hamburg, Lübeck, Kiel Germany; 13 Department of Medicine, University of Turku, Turku, Finland; 14 Division of Medicine, Turku University Hospital, Turku, Finland; 15 Department of Clinical Physiology, University of Tampere and Tampere University Hospital, Tampere, Finland; 16 Department of Clinical Chemistry, Fimlab Laboratories, School of Medicine, University of Tampere, Tampere, Finland; 17 Estonian Genome Center, University of Tartu, Tartu, Estonia; 18 Department of Epidemiology and Biostatistics, MRC-PHE Centre for Environment and Health, School of Public Health, Imperial College London, London, UK; 19 Research Centre of Applied and Preventive Cardiovascular Medicine, University of Turku, Turku, Finland; 20 Department of Clinical Physiology and Nuclear Medicine, Turku University Hospital, Turku, Finland; 21 Medical Research Council Integrative Epidemiology Unit at the University of Bristol, Bristol, UK; 22 School of Social and Community Medicine, University of Bristol, Bristol, UK

**Keywords:** Pregnancy, Trimesters, Postpartum, Metabolomics, Cytokines, Lipoprotein lipids, Fatty acids, Amino acids, Hormones, Inflammation, Metabolic networks

## Abstract

**Background:**

Pregnancy triggers well-known alterations in maternal glucose and lipid balance but its overall effects on systemic metabolism remain incompletely understood.

**Methods:**

Detailed molecular profiles (87 metabolic measures and 37 cytokines) were measured for up to 4260 women (24–49 years, 322 pregnant) from three population-based cohorts in Finland. Circulating molecular concentrations in pregnant women were compared to those in non-pregnant women. Metabolic profiles were also reassessed for 583 women 6 years later to uncover the longitudinal metabolic changes in response to change in the pregnancy status.

**Results:**

Compared to non-pregnant women, all lipoprotein subclasses and lipids were markedly increased in pregnant women. The most pronounced differences were observed for the intermediate-density, low-density and high-density lipoprotein triglyceride concentrations. Large differences were also seen for many fatty acids and amino acids. Pregnant women also had higher concentrations of low-grade inflammatory marker glycoprotein acetyls, higher concentrations of interleukin-18 and lower concentrations of interleukin-12p70. The changes in metabolic concentrations for women who were not pregnant at baseline but pregnant 6 years later (or vice versa) matched (or were mirror-images of) the cross-sectional association pattern. Cross-sectional results were consistent across the three cohorts and similar longitudinal changes were seen for 653 women in 4-year and 497 women in 10-year follow-up. For multiple metabolic measures, the changes increased in magnitude across the three trimesters.

**Conclusions:**

Pregnancy initiates substantial metabolic and inflammatory changes in the mothers. Comprehensive characterisation of normal pregnancy is important for gaining understanding of the key nutrients for fetal growth and development. These findings also provide a valuable molecular reference in relation to studies of adverse pregnancy outcomes.

**Electronic supplementary material:**

The online version of this article (doi:10.1186/s12916-016-0733-0) contains supplementary material, which is available to authorized users.

## Background

Pregnancy induces remarkable changes in maternal metabolism to support fetal demands [1, 2]. Maternal circulating glucose is an essential nutrient for the developing fetus [3] and during pregnancy women become increasingly insulin resistant, particularly from the second trimester onwards [4]. In the third trimester, maternal fasting insulin levels increase by over 30%, while fasting glucose concentrations decrease by about 10%, despite increased insulin resistance [1, 5]. In addition to glucose, there is evidence that circulating lipids and amino acids are also important nutrients for the fetus [1, 2]. During the third trimester, maternal circulating lipid concentrations dramatically increase, with triglycerides being elevated by approximately twofold, and total and low-density lipoprotein (LDL) cholesterol by some 30–50% [1, 5–7]. Maternal circulating amino acid concentrations are also suggested to be altered largely in response to increased protein synthesis for placental and fetal growth [8]. In addition, pregnancy triggers considerable changes in the maternal immune system, with circulating cytokines being reprogrammed to sustain the integrity of the allograft fetus [9].

Most of the changes in maternal metabolism and inflammatory status are considered to be normal physiological responses to support fetal growth and development. These changes typically return to pre-pregnancy states soon after delivery [2]. However, in some women the changes may be harmful and related to adverse pregnancy outcomes, for example, gestational diabetes, hypertensive disorders, and preterm birth [2, 10, 11]. These adverse pregnancy outcomes are associated with increased future risk of diabetes and cardiovascular disease both in mothers and offspring, though the extent to which these are causal or reflect pre-existing maternal risk is unclear [2]. Characterising the normal metabolic and inflammatory changes in pregnancy is crucial for gaining understanding of the key nutrients for normal fetal growth and development, and for identifying metabolic and inflammatory changes in pregnancy that might herald the risk of adverse outcomes. However, so far, most epidemiological studies on the metabolic effects of pregnancy have had very small numbers of individuals (typically only a few dozen pregnant women) and lack replication. The metabolic information is generally limited to standard lipids and glucose [1, 5], and studies on the effects of pregnancy on mothers’ systemic metabolism are lacking. The aim of this study is to comprehensively characterise the maternal systemic metabolism across a wide range of metabolic and inflammatory measures in both cross-sectional and longitudinal settings coupled with replication.

## Methods

### Study population

Serum metabolic profiles were quantified in three independent population-based cohorts from Finland. Samples for profiling were collected in 1997 for participants in the Northern Finland Birth Cohort 1966 (NFBC1966, *n* = 2963 women aged 31) [12, 13], in 2001 for those in the Cardiovascular Risk in Young Finns Study (YFS, *n* = 1239 women aged 24–39) [14], and in 1997 for those in the FINRISK1997 study (FINRISK1997, *n* = 2101 women aged 24–49) [15]. Overnight fasting samples were obtained for NFBC1966 and YFS, whereas semi-fasting samples (median fasting time 5 hours) were obtained for FINRISK1997. Further details of the study populations are provided in Additional file 1: Supplementary Methods. We excluded women whose information on pregnancy status at the time of metabolic profiling was missing (*n* = 136); women using oral contraception (*n* = 1279); and women with a fasting glucose ≥7 mmol/L (*n* = 33, of which two were pregnant), systolic blood pressure ≥140 mmHg, or diastolic blood pressure ≥90 mmHg (*n* = 595; 13 pregnant). After these exclusions, 4260 women, of whom 322 were pregnant (Table 1), were included in the study. A subset of 583 women from YFS attended a 6-year follow-up (baseline in 2001; follow-up in 2007) at which their clinical and pregnancy status were assessed and the metabolic profiling performed again. In these women prospective changes in their metabolic profiles, with respect to the change in their pregnancy status, were examined. Cytokine profiles were analysed for 2321 women from FINRISK1997 (1415 women, 69 pregnant) and YFS in 2007 (906 women, 35 pregnant).

**Table 1:** Characteristics of the study participants

Characteristics	NFBC1966		YFS		FINRISK1997	
	Non-pregnant	Pregnant	Non-pregnant	Pregnant	Non-pregnant	Pregnant
Number	1782	191	806	60	1350	71
Gestational age at blood sampling (week)	–	22 (13–29)	–	23 (15–30)	–	–
Age (year)	31 (0)	31 (0)	32 (5)	31 (5)	37 (7)	32 (5)
Weight (kg)	65 (13)	67 (11)	67 (13)	71 (12)	66 (12)	70 (13)
Waist (cm)	79 (11)	82 (12)	80 (11)	85 (10)	77 (10)	83 (13)
Hip (cm)	97 (8)	97 (8)	100 (9)	102 (7)	99 (8)	101 (9)
BMI (kg/m^2^)	24 (4)	24 (4)	24 (5)	26 (4)	25 (4)	26 (4)
Systolic blood pressure (mmHg)	117 (10)	116 (9)	111 (10)	109 (11)	119 (10)	114 (11)
Diastolic blood pressure (mmHg)	73 (8)	66 (10)	68 (8)	61 (9)	74 (8)	68 (9)
Smoking prevalence (%)	38	25	20	10	23	8
Alcohol usage (g/day)	2.1 (0.6–5.7)	1.0 (0.0–3.5)	3.3 (0.0–8.2)	0.0 (0.0–0.0)	1.8 (0.0–7.0)	0.0 (0.0–0.0)
Parity (n)	2 (1–2)	1 (0–2)	1 (0–2)	1 (0–2)	–	–

Values are mean (SD) for normally distributed and median (interquartile range) for skewed variables. Information on whether women have given birth before was not available for FINRISK1997. BMI: body mass index

We were not able to exclude breastfeeding women or those women who were pregnant with multiple babies because such information was not available in all the cohorts. However, in NFBC1966 where the information on the number of babies per pregnancy was available, there were only two pregnant women with a twin pregnancy and none with higher multiples. We therefore assume that the vast majority of the data in these population-based cohorts are on singleton pregnancies. Information on breastfeeding was available in YFS; 6.5% of non-pregnant women were breastfeeding. The metabolic associations with pregnancy were almost identical whether the data for breastfeeding women were excluded from those for non-pregnant women or not (Additional file 1: Figure S1).

### Information on pregnancy and covariates

Pregnancy status, parity, current smoking, and alcohol consumption were assessed by questionnaires. In YFS, the participants reported the gestational age (in weeks) at the time of blood sampling. In NFBC1966, the gestational age (in weeks) at the time of blood sampling was calculated based on a birth registry. Information on the gestational age was not available for the participants in FINRISK1997. Weight, height and blood pressure were assessed in clinics using established protocols.

In the primary analyses, we compared pregnant (*n* = 322) to non-pregnant women (*n* = 3938). In the secondary analyses, we compared pregnant women in different trimesters to non-pregnant women using data from NFBC1966 and YFS. Profiles for women in their first trimester (pregnancy length ≤12 weeks, *n* = 48), second trimester (pregnancy length >12 weeks and ≤28 weeks, *n* = 116), and third trimester (pregnancy length >28 weeks, *n* = 67) were compared to those of the non-pregnant women (*n* = 2588). Information on gestational age was missing for 20 pregnant women who were therefore excluded from these analyses.

### Molecular profiling

We analysed 124 biomarkers, including 87 metabolic measures and 37 cytokines. Eighty out of the 87 metabolic measures were quantified by a high-throughput serum nuclear magnetic resonance (NMR) metabolomics platform [16, 17]. These measures represent a broad molecular signature of systemic metabolism and cover multiple metabolic pathways, including lipoprotein lipids and subclasses, fatty acids, amino acids, and glycolysis-related metabolites. The NMR-based metabolic profiling has previously been used in multiple large-scale epidemiological and genetic studies [18–25] and the experimentation is described elsewhere [16, 17, 26]. The seven other metabolic measures assessed were high-sensitivity C-reactive protein (CRP); vitamin D; sex hormone-binding globulin (SHBG); and the hormones insulin, leptin, adiponectin and testosterone. Additionally, 37 cytokines were analysed using Bio-Rad’s Bio-Plex Assays. Details of these measurements are given in Additional file 1: Supplementary Methods.

### Statistical analyses

The metabolic and cytokine measures were log-transformed because their distributions were generally skewed (Additional file 1: Figure S2) and likely to result in non-normal residuals in the regression analyses. The measures were then scaled to standard deviations (SD) in each cohort. Due to the correlated nature of the data, we used principal component analysis to evaluate the appropriate number of independent tests for multiple testing correction [22, 24]. Sixty-one principal components explained over 99% of the variation in the molecular measures across all three cohorts (Additional file 1, Table S1). Therefore, *P* < 0.0008 (0.05/61) was used to infer statistical significance [22, 24].

For the cross-sectional analyses, a linear regression model was fitted for each outcome measure (concentration of a metabolic or a cytokine measure) using pregnancy status as the explanatory variable. Non-pregnant women were used as the reference group, so the association magnitudes denote the difference in each outcome measure between pregnant and non-pregnant women. Association magnitudes are reported in SD units to ease the comparison across multiple measures. Differences in the original units of the metabolic and cytokine measures and the percentage differences are given in Additional file 1: Supplementary Material. All three cohorts were analysed separately and the results combined using fixed-effect inverse variance-weighted meta-analysis after confirming the consistency across the cohorts. In the main analyses we adjusted for age. In the second set of models we additionally adjusted for body mass index (BMI), parity, mean arterial pressure (MAP), current smoking and alcohol consumption. Similar linear models were used to determine the metabolic differences between trimesters and non-pregnant women using data from NFBC1966 and YFS.

Those 583 women from the YFS cohort who had both baseline and 6-year follow-up data were classified as pregnant at follow-up (non-pregnant at baseline but pregnant at follow-up, *n* = 18), pregnant at baseline (pregnant at baseline but non-pregnant at follow-up, *n* = 44) and those non-pregnant at both time points (*n* = 519). Two women who were pregnant at both baseline and follow-up were excluded from the analysis. The 6-year changes in metabolic concentrations were compared between (1) women who were pregnant at follow-up, and (2) women who were pregnant at baseline, and women who were non-pregnant at both time points. The longitudinal models were adjusted for baseline age and further for the baseline parity and 6-year change in BMI, MAP, smoking and alcohol use. The longitudinal analyses were additionally replicated for 653 women who had 4-year follow-up data (from year 2007 to 2011 in YFS) and 497 women who had 10-year follow-up data (from year 2001 to 2011 in YFS). Details of these data are described in Additional file 1: Supplementary Methods.

## Results

The characteristics of the study participants are given in Table 1. The pregnant women, in comparison to those non-pregnant, tended to have lower diastolic blood pressure, smoke less and consume less alcohol.

### Lipoprotein-related measures in pregnancy

**Fig. 1:**
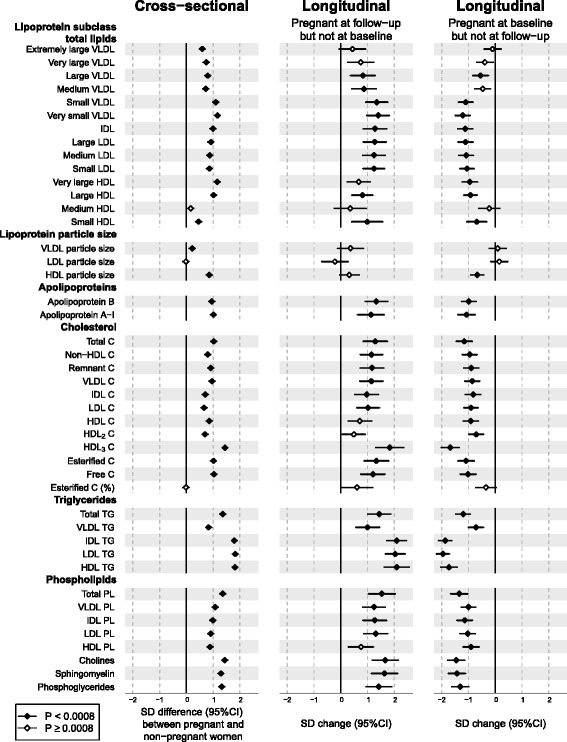
Cross-sectional and longitudinal associations between pregnancy and lipoprotein-related measures. *Left panel*, cross-sectional analyses in which pregnant women (*n* = 322) were compared to non-pregnant women (*n* = 3938). The associations were adjusted for age and meta-analysed for three Finnish population-based cohorts. *Middle panel*, longitudinal analyses in which women who were pregnant at follow-up (but not at baseline) (*n* = 18) were compared to women who were non-pregnant at both time points (*n* = 519). *Right panel*, longitudinal analyses in which women who were pregnant at baseline (but not at follow-up) (*n* = 44) were compared to women who were non-pregnant at both time points (*n* = 519). Longitudinal associations were adjusted for baseline age. *Open diamonds* indicate *P* ≥ 0.0008, *closed diamonds* indicate *P* < 0.0008. *C* cholesterol, *CI* confidence interval, *HDL* high-density lipoprotein, *IDL* intermediate-density lipoprotein, *LDL* low-density lipoprotein, *PL* phospholipids, *SD* standard deviation, *TG* triglycerides, *VLDL* very-low-density lipoprotein

Figure 1 (left panel) illustrates the cross-sectional associations between pregnancy and 44 lipoprotein-related measures. The metabolic differences between pregnant and non-pregnant women are given in SD units in Additional file 1: Table S2, in absolute physiological units in Additional file 1: Table S3 and as a percentage difference relative to non-pregnant women in Additional file 1: Table S4. Pregnancy was associated with increased levels of almost all lipoprotein-related measures (*P* < 0.0008 for 41 out of 44 measures meta-analysed). The association magnitudes were substantial, with a median of 0.9 SD unit increments (interquartile range 0.8–1.1 SDs), corresponding to a median of a 33% (interquartile range 25–51%) higher concentration in pregnant than in non-pregnant women. The increase in total lipids was similar across the smaller very-low-density (VLDL), intermediate-density (IDL) and low-density (LDL), and the larger high-density (HDL) lipoprotein subclasses. Pregnancy was positively associated with all cholesterol, triglyceride and phospholipid concentrations as well as with both apolipoprotein B and A-I. The differences for IDL, LDL and HDL triglycerides were approximately twofold in comparison to VLDL triglycerides. In contrast, differences in cholesterol and phospholipid concentrations in all these main lipoprotein fractions were broadly similar. The percentage of esterified cholesterol (of total cholesterol) did not differ between pregnant and non-pregnant women. Pregnancy was positively associated with the average size of VLDL and HDL particles but not with the average size of LDL particles.

### Fatty acids in pregnancy

**Fig. 2:**
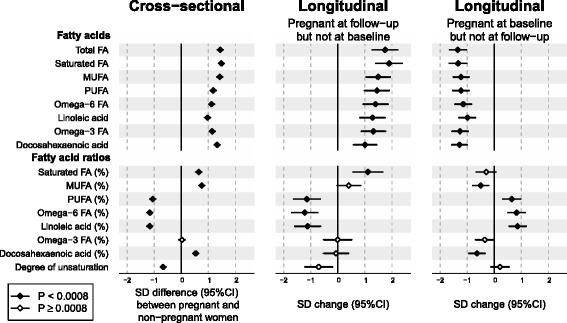
Cross-sectional and longitudinal associations between pregnancy and fatty acids. The study design is as explained in the legend for Fig. 1. The percentage sign (*%*) refers to the proportion of an individual measure of the total fatty acids. *Open diamonds* indicate *P* ≥ 0.0008, *closed diamonds* indicate *P* < 0.0008. *CI* confidence interval, *FA* fatty acids, *MUFA* monounsaturated fatty acids, *PUFA* polyunsaturated fatty acids, *SD* standard deviation

Figure 2 (left panel) displays the cross-sectional associations between pregnancy and multiple fatty acid measures. The circulating concentrations of all fatty acids were considerably increased during pregnancy, ranging approximately between 1.0 and 1.5 SDs, being equivalent to 24–56% higher concentrations relative to non-pregnant women. However, the proportions of individual fatty acids (relative to total fatty acids) exhibited a diverse association pattern. The proportions of saturated and monounsaturated fatty acids as well as the proportion of docosahexaenoic acid (DHA) were increased. In contrast, the proportion of omega-6 fatty acids, including linoleic acid, were decreased.

### Metabolites, inflammatory markers and hormones in pregnancy

**Fig. 3:**
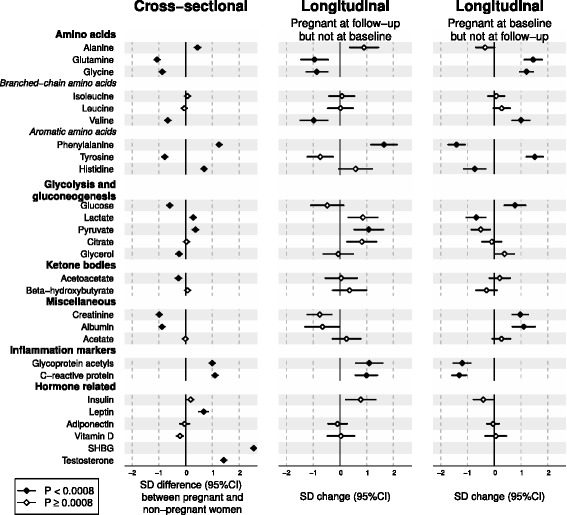
Cross-sectional and longitudinal associations between pregnancy and metabolic, inflammatory and hormonal measures. The study design is as explained in the legend for Fig. 1. *Open diamonds* indicate *P* ≥ 0.0008, *closed diamonds* indicate *P* < 0.0008. *CI* confidence interval, *SD* standard deviation, *SHBG* sex hormone-binding globulin

Figure 3 (left panel) shows the concentration differences in multiple circulating biomarkers between pregnant and non-pregnant women. The amino acids displayed a heterogeneous association pattern: strong positive associations for alanine, phenylalanine and histidine; negative associations for glutamine, glycine, valine and tyrosine; and null associations for isoleucine and leucine. Glucose, glycerol and acetoacetate concentrations were modestly decreased but lactate and pyruvate increased in the pregnant women. Creatinine and albumin concentrations were decreased and those of the low-grade inflammatory markers, glycoprotein acetyls (GlycA) [27] and CRP, increased. Of the hormones, testosterone and leptin concentrations were increased, as was that of SHBG, which displayed the strongest association across the molecular profile (2.5 SDs; equivalent to 483% higher concentration in pregnant women than in the non-pregnant women).

The associations with lipoprotein-related measures, fatty acids, low-molecular-weight metabolites, inflammatory markers and hormones were highly consistent across the three cohorts (Additional file 1: Figure S3). The results remained very similar with further adjustments for parity, BMI, MAP, smoking and alcohol (Additional file 1: Figure S4). To assess how much the metabolic signature of pregnancy could reflect high concentrations of circulating lipids, it was compared to the metabolic association patterns of higher non-VLDL-triglycerides (the sum of IDL, LDL and HDL triglycerides) and higher total fatty acids in non-pregnant women (Additional file 1, Figure S5). These analyses support an interpretation that many of the metabolic changes seen are likely specific for the pregnancy status.

### Correlation network analysis

Multivariate correlation network analyses were conducted to assess potential differences in metabolic associations between pregnant and non-pregnant women; these analyses and the results are described in detail in Additional file 2. Briefly, a higher degree of pair-wise correlations was observed in pregnant (mean Spearman |r| = 0.39) compared to non-pregnant women (mean |r| = 0.29). A pronounced node difference was noted for SHBG, together with triglycerides in multiple lipoprotein categories (‘Triglyceride enrichment’ community), alanine and phenylalanine (‘Amino acid’) and various fatty acids (‘Fatty acid composition’). These nodes shared a similar pattern of higher inter-community connectivity for the pregnant than for the non-pregnant women. In general, the correlation network analyses suggest that pregnancy is acting as a metabolic stressor that amplifies intrinsic patterns of metabolism.

### Metabolic changes in response to change in pregnancy status

**Fig. 4:**
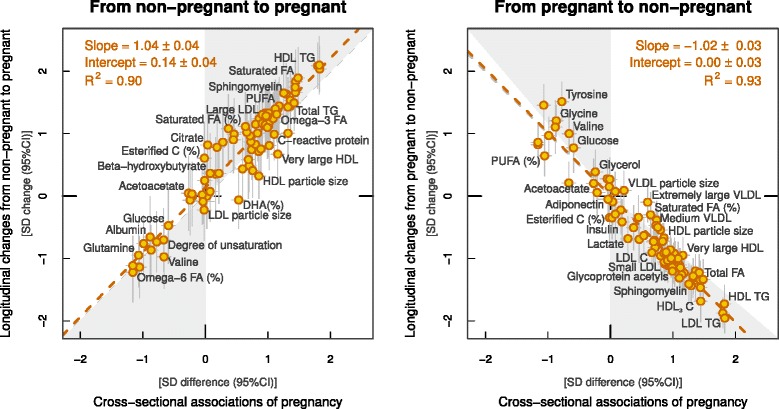
Correlations between cross-sectional and longitudinal metabolic associations of pregnancy. *Left panel*, linear fit to summarise the correspondence between cross-sectional (pregnant women compared to non-pregnant women) and longitudinal associations for women pregnant at follow-up but not at baseline (in comparison to women non-pregnant at both time points). *Right panel*, linear fit to summarise the correspondence between cross-sectional and longitudinal associations for women pregnant at baseline but not at follow-up (in comparison to women non-pregnant at both time points). Each *point* represents a single metabolic measure. *Horizontal* and *vertical grey lines* denote 95% CIs for the cross-sectional and longitudinal associations, respectively. The *grey shaded areas* serve to guide the eye for the slope. A linear fit for the overall correspondence summarises the match between cross-sectional and longitudinal associations, with *R* ^2^ denoting the goodness of fit. A slope of ±1 and *R* ^2^ = 1 would strongly support the causal effects of pregnancy on the metabolic measures. *C* cholesterol, *CI* confidence interval, *DHA* docosahexaenoic acid, *FA* fatty acids, *HDL* high-density lipoprotein, *IDL* intermediate-density lipoprotein, *LDL* low-density lipoprotein, *PL* phospholipids, *PUFA* polyunsaturated fatty acids, *SD* standard deviation, *TG* triglycerides, *VLDL* very-low-density lipoprotein

Figures 1, 2 and 3 (middle and right panels) illustrate six-year changes in 84 metabolic measures in response to change in pregnancy status (follow-up data were missing for leptin, SHBG and testosterone). In the middle panels, the metabolic changes in women who were pregnant at follow-up but not at baseline were compared to those of women who were non-pregnant at both time points. In the right panels, the metabolic changes in women who were pregnant at baseline but not at follow-up were compared to those of women who were non-pregnant at both time points. Substantial metabolic changes were observed for women who were pregnant at follow-up (middle panel); the association magnitudes were highly similar to those observed in the cross-sectional setting (left panel). Pronounced metabolic changes were also observed for women who were pregnant at baseline (right panel); the absolute association magnitudes again matched the cross-sectional setting, but they were in the opposite direction. The overall consistency between the longitudinal metabolic changes in women pregnant at follow-up and the cross-sectional metabolic differences followed a straight line with a slope of 1.04 ± 0.04 (R^2^ = 0.90; Fig. 4, left panel). Similarly, the magnitudes of the metabolic associations for women pregnant at baseline and those of cross-sectional associations also fell in a straight line with R^2^ = 0.93 but with a negative slope of −1.02 ± 0.03 (Fig. 4, right panel).

Differences in the point estimates were noted for DHA (%) in the longitudinal analysis of women who were pregnant at follow-up but not at baseline, in comparison with the other longitudinal analysis and cross-sectional analysis. However, the confidence intervals of these analyses overlapped, suggesting consistency of the results. Larger longitudinal studies would be required to ascertain the relevance of these findings on DHA% in pregnancy. The longitudinal associations remained similar when further adjusted for baseline parity and 6-year change in BMI, MAP, smoking and alcohol consumption (Additional file 1: Figures S6 and S7). To assess the consistency of these longitudinal associations in the YFS cohort, we further compared the results from the 6-year follow-up to those from 4-year (from year 2007 to 2011) and 10-year follow-up (from year 2001 to 2011). The longitudinal associations were highly consistent across the 4-year, 6-year and 10-year follow-up (Additional file 1: Figure S3).

### Metabolic profile during trimesters

**Fig. 5:**
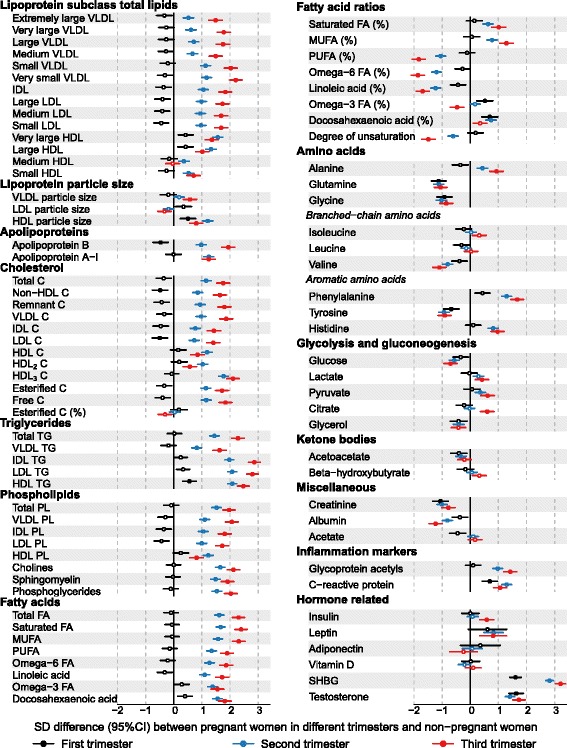
Cross-sectional associations between pregnancy trimesters and metabolic measures. Pregnant women in their first (n = 48), second (n = 116) and third trimester (n = 67) were compared to non-pregnant women (n = 2588). Associations were adjusted for age and meta-analysed for NFBC1966 and YFS. *Open circles* indicate *P* ≥ 0.0008, *closed circles* indicate *P* < 0.0008. *C* cholesterol, *CI* confidence interval, *FA* fatty acids, *HDL* high-density lipoprotein, *IDL* intermediate-density lipoprotein, *LDL* low-density lipoprotein, *PL* phospholipids, *MUFA* monounsaturated fatty acids, *PUFA* polyunsaturated fatty acids, *SD* standard deviation, *SHBG* sex hormone-binding globulin, *TG* triglycerides, *VLDL* very-low-density lipoprotein

Figure 5 shows cross-sectional concentration differences in 87 metabolic measures between pregnant women in different trimesters and non-pregnant women (Additional file 1: Table S5). The differences in SD units, in absolute physiological units and as percentages relative to non-pregnant women are given in Additional file 1: Tables S2, S3 and S4, respectively. During the first trimester many circulating metabolic concentrations were similar to those in non-pregnant women, although some clear differences were already observable: for example, glutamine, creatinine, SHBG and testosterone displayed over 1 SD metabolic differences. Women in their second or third trimesters of pregnancy had large metabolic aberrations in comparison to non-pregnant women, with similar association patterns as those described earlier (Figs. 1, 2 and 3) when all trimesters were combined. There was a clear trend of increasing metabolic differences in most lipoprotein-related measures across the trimesters, with median percentage difference −9% (interquartile range −13% to 0%) for the first trimester, 34% (interquartile range 28–51%) for the second trimester, and 59% (interquartile range 39–100%) for the third trimester. However, HDL-related measures were similar during the second and third trimesters. The association pattern across the three trimesters for most of the absolute concentrations of fatty acids followed that of the apolipoprotein B-containing lipoprotein subclasses. However, exceptions were observed for omega-3 fatty acids and DHA, for which the association pattern across the three trimesters resembled that of the large HDL subclasses and apolipoprotein A-I. The differences in the majority of the proportions of the fatty acids also had the tendency to be largest during the third trimester. The circulating concentrations of multiple low-molecular-weight metabolites, albumin, inflammatory markers and hormone-related measures were broadly similar during the second and third trimesters. The metabolic associations during the trimesters were highly consistent in NFBC1966 and YFS (Additional file 1: Figure S8).

### Metabolic retention of pregnancy

The metabolic retention of pregnancy was evaluated at various times postpartum (calculated as the duration from the delivery to the blood sampling). These analyses were done in NFBC1966 (serum samples collected at 1997) utilising the birth registry data from 1995 to 1997. Women postpartum up to 2 months (*n* = 28), over 2 and up to 4 months (*n* = 46), over 4 and up to 6 months (*n* = 42) and over 6 and up to 12 months (*n* = 123) were compared to those 1–3 years postpartum (*n* = 389). There were clear trends of metabolic normalisation after the delivery. Within 3–6 months postpartum, most of the metabolic disturbances were back to levels similar to those in women 1–3 years postpartum (Additional file 1: Figure S9). We only had the birth registry data for NFBC1966 so we could not specify the delivery dates for all the non-pregnant women in this study. However, according to the NFBC1966 data, only 6.5% of women had given birth within 6 months prior to blood sampling. When these women were excluded from the non-pregnant women in the analyses, the metabolic associations with pregnancy were almost identical (Additional file 1: Figure S10).

### Cytokine profile in pregnancy

The cross-sectional associations of pregnancy with 37 cytokines are illustrated in Additional file 1: Figure S9. Among interleukins, positive associations were found with IL-1ra and IL-18 and negative associations with IL-12p70. In addition, pregnant women had lower levels of TRAIL, MCP-1, eotaxin and VEGF and higher levels of HGF and βNGF than non-pregnant women. The abbreviations of the cytokines are explained in the caption for Additional file 1: Figure S11.

## Discussion

Here we have comprehensively characterised the effects of pregnancy on maternal systemic metabolism across a wide range of metabolic and inflammatory measures in large numbers of women. These findings are important for a broader metabolic understanding of the physiological changes caused by pregnancy per se that are likely to be key to normal fetal growth and development. They also provide a valuable molecular reference in relation to studies of adverse pregnancy outcomes [2]. The cross-sectional associations in three population-based studies consistently showed that pregnancy was associated with a wide range of metabolic pathways, including those for lipoproteins, fatty acids, amino acids, inflammatory markers and hormones. The metabolic associations were pronounced, with magnitudes during the third trimester being generally 1–3 SDs different from the non-pregnant women. The exceptionally large metabolic effects, together with the consistent results from longitudinal analyses, indicate that the metabolic aberrations arise as a direct result of pregnancy [28]. The metabolic changes increased in magnitudes for many metabolic measures across the trimesters, and the changes caused by pregnancy generally normalised within 3–6 months postpartum.

Metabolic adaptation to pregnancy has been widely studied with respect to glucose metabolism and standard lipids [1, 5–7]. Observational studies have reported that decreased circulating glucose and increased triglycerides, LDL and HDL cholesterol as well as insulin are associated with pregnancy [1, 5–7]. Our results here confirm these metabolic changes in pregnant women and their change patterns and effect sizes with respect to the trimesters. These findings, together with the results for leptin [29, 30], SHBG and testosterone [31], serve as good positive controls for the novel metabolomics approach taken in this work.

The most pronounced metabolic changes during pregnancy were observed in the circulating concentrations of IDL, LDL and HDL triglycerides. In these lipoprotein fractions, the increase in triglycerides was more pronounced than the increase in cholesterol and phospholipids, suggesting triglyceride-enrichment of these circulating lipoprotein particles in pregnancy [32], particularly during the second and third trimesters. In contrast, in the VLDL fraction, all these key lipid constituents increased almost in parallel with each other. Similar results have been reported previously in small longitudinal studies [32, 33], with a further suggestion that the triglyceride-enrichment in various lipoprotein fractions would be related to oestrogen-induced enhanced VLDL production and decreased activity of lipoprotein lipase and hepatic lipase during pregnancy. Maternal triglycerides are widely considered to be an important nutrient for fetal growth. Various observational studies have shown that higher maternal triglyceride concentrations are associated with larger neonatal fat mass and higher birth weight [34–37]. However, a recent Mendelian randomisation analysis challenged the notion that maternal triglycerides would be causally related to birth weight [3]. The triglyceride-enrichment in IDL, LDL and HDL observed here suggests a specific role of maternal triglycerides in different lipoprotein fractions. This points to a potential value of further research to assess the causal relations between maternal triglycerides carried in different lipoprotein particles and birth weight.

Fatty acids are required by the developing fetus to support rapid cellular growth and activity [38]. Our results showed that total circulating maternal fatty acid concentration gradually increased during pregnancy, in line with the overall increase in lipoprotein lipids. The fatty acid composition, relative to total fatty acids, was also largely modified during pregnancy. Similar findings for maternal fatty acids and their proportions in plasma and plasma phospholipids have been reported previously [39, 40]. DHA may be of particular importance to fetal brain and retinal development [38]. Omega-3 fatty acid supplementation during pregnancy appears to increase maternal and fetal DHA concentration [38], but meta-analysis of randomised controlled trials suggests that omega-3 fatty acid supplementation, in addition to an adequate omnivore intake, can only cause a modest increase in birth weight and seems to have no effects on birth length or head circumference [41, 42]. The potential effects of maternal omega-3 supplementation during pregnancy on cognitive and visual development in early childhood remain inconclusive [43].

Maternal amino acids are considered to be key determinants for fetal growth [1]. Nevertheless, information on maternal amino acids during pregnancy is sparse and comes from studies with only small numbers of participants [8, 44, 45]. A small cross-sectional study (with some 10 pregnant women in each trimester) found that most amino acid concentrations decreased during pregnancy [44]. However, our cross-sectional and longitudinal findings here consistently indicate that amino acids actually display a complex association pattern, with some being markedly elevated (alanine, phenylalanine and histidine), some depleted (glutamine, glycine, valine and tyrosine) and some (isoleucine and leucine) not responding to pregnancy. The association of pregnancy with branched-chain and aromatic amino acid concentrations did not follow the pattern of consistent elevations previously seen with obesity and insulin resistance [18, 19, 46]. Thus, it is unlikely that the weight gain or the development of insulin resistance during pregnancy underlie these changes in maternal amino acids. The complex associations of pregnancy with amino acids are likely to reflect the intricate molecular-specific interplay between maternal, placental and fetal metabolism [8]. For example, there is evidence that the concentrations of some amino acids are generally higher in umbilical cord plasma than in maternal circulation at delivery [8]. Amino acids cross the placenta via a combination of multiple active transport systems [47]. Dietary interventions during pregnancy have shown that an increase in maternal circulating amino acid concentrations does not appear to affect their circulating concentrations in the umbilical cord or in the neonate [8]. These findings suggest a robust placental transporting system in adequately nourished mothers. However, in the present study we are unable to explore these maternal–fetal interactions.

The maternal immune system is challenged by the conflicting demands of maintaining robust immune reactivity to protect both the mother and the fetus while at the same time tolerating highly immunogenic alloantigens to sustain fetal integrity [48]. We found that concentrations of the low-grade inflammatory markers CRP and GlycA were clearly increased during pregnancy, suggesting the up-regulation of systemic maternal inflammation. The new inflammatory marker, GlycA, which is quantified via the serum NMR metabolomics platform, has recently been shown to be associated with both acute-phase and chronic inflammation, as well as being related to increased neutrophil activities [27, 49]. Elevation of GlycA during pregnancy may thereby suggest an enhancement of maternal innate immunity. With respect to adaptive immunity, previous studies have suggested that normal pregnancy polarises the immune response towards the T helper 2 (Th2) response [9, 48, 50]. This notion is consistent with our findings of increased circulating IL-18 but decreased levels of IL12p70, which are important regulators of the Th2/Th1 balance [9, 50].

Metabolic and immunological changes in the mothers predominantly occur to the benefit of the fetus. However, some of these changes may be unfavourable to the mothers. Hypertriglyceridaemia, elevated LDL and remnant cholesterol, insulin resistance, and up-regulated inflammation during pregnancy are all features related to the increased risk of cardiometabolic diseases. In addition, our results here indicate that multiple new biomarkers for cardiovascular diseases [17, 20] show unfavourable changes during pregnancy; these include elevated concentrations of phenylalanine and the increased proportion of monounsaturated and decreased proportions of omega-6 fatty acids. Although we found that pregnancy-related metabolic alterations generally normalise soon after delivery (Additional file 1: Figure S9), there is some evidence that repeated pregnancies might predispose women to higher cardiovascular risk at older age [51, 52]. It has been reported that the association between parity and cardiovascular risk is J-shaped, with two births representing the lowest risk [51, 52]. Compared to women with two births, women with five or more births had a 66% higher risk of cardiovascular disease, the association remaining robust when adjusting for socioeconomic status and pregnancy complications [52]. However, it remains unclear whether the association between parity and cardiovascular risk indicates a causal relationship [2].

Oral contraceptives are used to prevent pregnancy by mimicking the reproductive hormone status of natural pregnancy through the supplementation of a combination of oestrogen and progestin (combined oral contraceptive pills; COCPs) or progestin-only contraceptives (POCs). Understandably, this simple mimic cannot replicate the complex physiological effects of natural pregnancy and the multilevel interactions between the mother, the placenta and the fetus. This is in line with our findings that there is little correspondence in the pattern of cytokine changes in pregnant women and those using COCPs [53]. Interestingly, however, the pattern of metabolic changes for lipoprotein measures, fatty acids, amino acids and low-grade inflammatory markers during pregnancy has a strong resemblance to the one caused by COCPs. Though the absolute metabolic changes are stronger in the case of pregnancy, the direction of change is generally consistent with that in women who use COCPs. On the other hand, the metabolic effects of POCs are weak or negligible [53]. These findings imply that oestrogen plays a key role in the regulation of maternal metabolism.

Owing to the population-based design of our cohorts, we were not able to look at how the circulating metabolic measures relate to potential pregnancy complications or perinatal outcomes. However, it is an important start to examine the metabolic consequences of normal pregnancy across a wide range of metabolic and inflammatory measures. We excluded women (whether pregnant or not) who had high fasting glucose or high blood pressure, at levels indicative of diabetes or hypertension, respectively. While we cannot be certain that we have excluded all cases of gestational diabetes or hypertensive disorders of pregnancy, the nature of general population studies suggests that our results reflect changes in normal pregnancy. The strengths of this study include extensive molecular profiling of systemic metabolism with replication across three independent population-based cohorts. The highly consistent metabolic findings from multiple longitudinal analyses (4, 6 and 10 years of follow-up) provide compelling additional evidence that the metabolic aberrations are a direct result of pregnancy. We also acknowledge that the trimester analyses are cross-sectional and the pregnancy status is assessed via questionnaires. This may slightly affect the metabolic associations during the first trimester, since some women may not be aware of being pregnant in their early pregnancy and thus report themselves as non-pregnant. Nevertheless, this information bias would be minimal in the case of middle and late pregnancy. Importantly, the extraordinarily large association magnitudes and gradual increases in effect sizes across the trimesters, together with the replication in individual cohorts, strongly suggest that the effect of confounding is minimal.

## Conclusions

This work characterises the effects of pregnancy on maternal metabolism across a wide range of metabolic and inflammatory measures. The findings are important for a broader metabolic understanding of the physiological changes caused by pregnancy per se and for assessing the metabolic milieu related to normal fetal growth and development. The metabolic effects of pregnancy are exceptionally large, gradually increase across the trimesters, and generally normalise within 3–6 months postpartum. These findings provide a comprehensive foundation to the systemic molecular understanding of maternal metabolism during pregnancy.

## Open Peer Review Reports

The authors’ response to reviewers is available as Additional file 3.

## Additional files

Additional file 1: Supplementary materials including detailed information on study populations, generalised data supporting our findings, and results from sensitivity analyses. (DOCX 2656 kb)

Additional file 2: Correlation network analysis. (PDF 7311 kb)

Additional file 3: Authors’ response to reviewers. (PDF 7434 kb)

## Abbreviations

BMI: Body mass index
COCP: Combined oral contraceptive pill
CRP: C-reactive protein
DHA: Docosahexaenoic acid
GlycA: Glycoprotein acetyls
HDL: High-density lipoprotein
IDL: Intermediate-density lipoprotein
LDL: Low-density lipoprotein
MAP: Mean arterial pressure
NFBC1966: Northern Finland Birth Cohort 1966
NMR: Nuclear magnetic resonance
POC: Progestin-only contraceptives
SD: Standard deviation
SHBG: Sex hormone-binding globulin
Th2: T helper 2.
VLDL: Very-low-density lipoprotein
YFS: Cardiovascular Risk in Young Finns Study The abbreviations of the cytokines are available in the caption of Figure S12 (Additional file 1).
