# Supplementary material for: Metabolic profiling of pregnancy: cross-sectional and longitudinal evidence
Source: BMC Med. 2016 Dec 13;14:205. doi: 10.1186/s12916-016-0733-0 (PMC5153817; doi:10.1186/s12916-016-0733-0)
Supplement: Supplementary file 1 — Supplementary materials including detailed information on study populations, generalised data supporting our findings, and results from sensitivity analyses. (DOCX 2656 kb) [file 12916_2016_733_MOESM1_ESM.docx]

**Supplementary Material**

Wang et al.

Metabolic Profiling of Pregnancy: Cross-sectional and Longitudinal Evidence

**Supplementary Methods**

Study populations

**Supplementary Tables**

Table S1. Details for the principal component analysis (PCA).

Table S2. Cross-sectional metabolic differences associated with pregnancy and trimesters in standard-deviation units.

Table S3. Cross-sectional metabolic differences associated with pregnancy and pregnancy trimesters in absolute concentration units.

Table S4. Cross-sectional metabolic differences associated with pregnancy and pregnancy trimesters in relative to non-pregnant women.

Table S5. Characteristics of the study participants during the pregnancy trimesters.

**Supplementary Figures**

Figure S1. Cross-section associations with and without the exclusion of breastfeeding women in YFS.

Figure S2. Distributions of metabolic measures quantified via the NMR metabolomics platform in the three Finnish cohorts.

Figure S3. Replication of cross-sectional and longitudinal associations between pregnancy and 87 metabolic measures.

Figure S4. Cross-sectional associations between pregnancy and 87 metabolic measures with or without further adjustment of parity, BMI, smoking, alcohol usage and mean arterial pressure.

Figure S5. Metabolic differences associated with pregnancy compared to those associated with higher non-VLDL-TG and higher total fatty acids (FA).

Figure S6. Longitudinal associations for women pregnant at follow-up with further adjustments.

Figure S7. Longitudinal associations for women pregnant at baseline with further adjustments.

Figure S8. Cross-sectional associations between the pregnancy trimesters and 87 metabolic measures in individual cohorts.

Figure S9. Cross-sectional associations of postpartum length with 85 metabolic measures.

Figure S10. Cross-sectional associations with and without the exclusion of women postpartum 0-6 months.

Figure S11. Cross-sectional associations between pregnancy and 37 cytokines.

**Supplementary Methods**

Study populations

*The Northern Finland Birth Cohort of 1966* (NFBC1966) was initiated to study factors affecting preterm birth and subsequent morbidity in the two northernmost provinces in Finland. It included 12 058 children born alive, comprising 96% of all births during 1966 in the region [1,2]. Data collection in 1997 included clinical examination and serum sampling at the age of 31 years for 6007 individuals. Data from this time point are analyzed in the present study. Attendees in the field study at age 31 were representative of the original cohort [1]. NMR-based metabolomics were measured for 2963 women with serum sample available, of which 96% were based on over-night fasting serum samples [3]. Among these, 2841 women also had information on pregnancy status. Women using oral contraception (n=651), and those with a fasting glucose ≥7mmol/L (n=12), systolic blood pressure ≥140 mmHg or diastolic blood pressure ≥90 (n=205), at the time of metabolic profiling were excluded. Following these exclusions 1973 women, of whom 191 were pregnant, were included in this study.

Pregnancy status, parity, current smoking and average alcohol use were assessed from questionnaires. Gestational age (in weeks) for pregnant women was calculated based on a birth registry: (entire pregnancy duration in days – (date of birth delivery – date of blood sampling))/7. Blood pressure was measured using a mercury sphygmomanometer. Plasma insulin, vitamin D, sex hormone-binding globulin (SHBG) and high-sensitivity C–reactive protein (CRP) were measured by standard clinical chemistry assays. Testosterone was measured by mass spectrometry. Informed written consent was obtained from all participants, and the research protocols were approved by the Ethics Committee of Northern Ostrobothnia Hospital District, Finland.

*The Cardiovascular Risk in Young Finns Study* (YFS) was designed to study associations of childhood risk factors to cardiovascular disease in adulthood [4]. The baseline study conducted in 1980 included 3596 children and adolescents aged 3–18 years. NMR-based metabolomics data were measured for 2247, 2160 and 2040 participants who had overnight fasting serum samples collected in 2001-, 2007- and 2011-survey, respectively. These individuals were representative of the original cohort [4]. The same exclusion criterions as those used in NFBC1966 were applied in each of the three YFS surveys. In the cross-sectional analysis, 866 women from YFS at 2001 were used (pregnant/non-pregnant=60/806). In the longitudinal analysis, the primary results were calculated from the 6-year follow-up from 2001 to 2007 (n= 583). To validate the longitudinal consistency, these results were further compared to those calculated from the 4-year follow-up from 2007 to 2011 (n=653) and 10-year follow-up from 2001 to 2011 (n=497). All those women used in the cross-sectional and longitudinal analyses had metabolic profiling measured and had information on pregnancy status. In addition, 906 women from YFS in 2007, of whom 35 were pregnant, also had cytokine panels measured (detailed description for cytokine profiling given below).

Pregnant status, gestational age, parity, current smoking and average alcohol usage were assessed by questionnaires. Blood pressure was measured using a random-zero sphygmomanometer. The following biomarkers were measured by standard clinical chemistry assays: high-sensitivity CRP, insulin, leptin, adiponectin, vitamin D, SHBG and testosterone. CRP and the six hormone-related measures were all measured for YFS in 2001. All but leptin, SHBG and testosterone were measured for YFS in 2007. Only CRP and insulin were measured for YFS in 2011. All participants gave written informed consent, and the study was approved by the ethics committees of each five participating medical university study sites.

*The FINRISK 1997 Study* (FINRISK1997) was conducted to monitor the health of the Finnish population among persons aged 24–74 at recruitment [5]. In total, 8444 individuals were recruited to represent the middle-aged population of the study areas. NMR-based metabolomics were measured for 3829 women who had the serum samples collected. The median fasting time was 5h (interquartile range 4–6h). After the same exclusion criterion as applied in NFBC1966, 1421 women (pregnant/non-pregnant=71/1350) who had the metabolic profile and information on the pregnancy status were included in this study. In addition, 1415 women (pregnant/non-pregnant=69/1346) also had cytokine profiles measured using the same technology as in YFS (details given below).

Pregnancy status, current smoking and average use of alcohol were assessed by questionnaires. Blood pressure was measured using a mercury sphygmomanometer. The following circulating biomarkers were assayed by standard clinical chemistry assays and analysed in the present study: insulin, leptin, adiponectin, vitamin D, testosterone, and high-sensitivity CRP. Participants gave written informed consent and the FINRISK study was approved by the Coordinating Ethical Committee of the Helsinki and Uusimaa Hospital District.

Cytokine profiling

Cytokine profiling was performed in FINRISK1997 and YFS. In total, 1415 women from FINRISK1997 (of whom 69 were pregnant) and 906 women from YFS (of whom 35 were pregnant), who had the cytokine data and information on pregnancy status, were used in the cross-sectional analysis.

YFS: Total of 48 cytokines were measured for 2200 individuals in the 2007 follow-up survey using Bio-Rad’s premixed Bio-Plex Pro Human Cytokine 27-plex Assay and 21-plex Assay, and Bio-Plex 200 reader with Bio-Plex 6.0 software [6]. The assays were performed according to manufacturer’s instructions, except, that the amount of beads, detection antibodies and streptavidin-phycoerythrin conjugate were used with 50% lower concentrations than recommended by the manufacturer. Only measures within the cytokine-specific detection range were included in the analyses. Low absolute concentrations of several cytokines (with respect to the sensitivity of the method) complicate their quantification. The Bio-Rad analyser program fitted the measured light signals from the individual samples to the standard curves generated with recombinant cytokines for each cytokine on each 96-well plate using a five-parameter logistic regression. Due to the non-linear standard curves, the upper and lower detection limits are calculated plate-wise, so that they corresponded with “asymptotic” concentrations representing fluorescent intensity 2% above lower and 2% below upper asymptote of the calibration curve. If more than 50% of the observations corresponded to the asymptotic concentrations or were missing (i.e., below the detection limit) for a particular measure, it was excluded from further analyses. This resulted in 11 cytokine measures to be excluded; 37 measures were subsequently used in the further analyses.

FINRISK: The same Bio-Plex assays were used to quantify the cytokines as in YFS. Nineteen of the 37 measures that were analyzed in YFS were also available in FINRISK1997.

**References**

1. Järvelin M-R, Sovio U, King V, Lauren L, Xu B, McCarthy MI, et al. Early life factors and blood pressure at age 31 years in the 1966 northern Finland birth cohort. Hypertension 2004;44:838–46.

2. Männistö T, Mendola P, Vääräsmäki M, Järvelin M-R, Hartikainen A-L, Pouta A, et al. Elevated blood pressure in pregnancy and subsequent chronic disease risk. Circulation 2013;127:681–90.

3. Würtz P, Mäkinen V-P, Soininen P, Kangas AJ, Tukiainen T, Kettunen J, et al. Metabolic signatures of insulin resistance in 7,098 young adults. Diabetes 2012;61:1372–80.

4. Raitakari OT, Juonala M, Rönnemaa T, Keltikangas-Järvinen L, Räsänen L, Pietikäinen M, et al. Cohort profile: the cardiovascular risk in Young Finns Study. Int J Epidemiol 2008;37:1220–6.

5. Jousilahti P, Laatikainen T, Peltonen M, Borodulin K, Männistö S, Jula A, et al. Primary prevention and risk factor reduction in coronary heart disease mortality among working aged men and women in eastern Finland over 40 years: population based observational study. BMJ 2016;352:i721.

6. Ritchie SC, Würtz P, Nath AP, Abraham G, Havulinna AS, Fearnley LG, et al. The Biomarker GlycA Is Associated with Chronic Inflammation and Predicts Long-Term Risk of Severe Infection. Cell Systems 2015;1:293–301.

7. Kujala UM, Mäkinen V-P, Heinonen I, Soininen P, Kangas AJ, Leskinen TH, et al. Long-term leisure-time physical activity and serum metabolome. Circulation 2013;127:340–8.

8. Wang Q, Kangas AJ, Soininen P, Tiainen M, Tynkkynen T, Puukka K, et al. Sex hormone-binding globulin associations with circulating lipids and metabolites and the risk for type 2 diabetes: observational and causal effect estimates. Int J Epidemiol 2015;44:623–37.

9. Wang Q, Würtz P, Auro K, Morin Papunen L, Kangas AJ, Soininen P, et al. Effects of hormonal contraception on systemic metabolism: cross-sectional and longitudinal evidence. Int J Epidemiol 2016;dyw147.

**Table S1. Details for the principal component analysis (PCA).**

| **Cohort** | **Sample size** | **Maximum number of variables available** | **Number of PCs explains at least 99% of variation in the data** |
| --- | --- | --- | --- |
| NFBC 1966 | 1349 | 85  (metabolic measures) | 34 |
| YFS  (2007 survey) | 1133 | 121  (84 metabolic measures + 37cytokines) | 61 |
| FINRISK1997 | 2872 | 105  (86 metabolic measures + 19cytokines) | 50 |

The rationale in defining the number of independent tests via PCA has been discussed previously [7,8]. The PCA was performed on each individual cohort. In each cohort, all women who had complete data for the maximum number of variables were used in the analysis. The numbers of samples and variables are listed for the individual cohorts. Since the number of available variables largely varied across the cohorts, the number of PCs necessary to explain at least 99% of variation in the data also varied. As YFS contained basically the complete set of variables, we chose 61 as the number of independent tests. Therefore, P values less than (0.05/61) were considered statistically significant after multiple testing correction.

**Table S2. Cross-sectional metabolic differences associated with pregnancy and trimesters in standard-deviation units.**

| **Metabolic measures** | **SD difference**  **associated with**  **pregnancy**  **Beta [95%CI]; P values** | **SD difference**  **associated with**  **first trimester**  **Beta [95%CI]; P values** | **SD difference**  **associated with**  **second trimester**  **Beta [95%CI]; P values** | **SD difference**  **associated with**  **third trimester**  **Beta [95%CI]; P values** |
| --- | --- | --- | --- | --- |
| **Lipoprotein subclass total lipids** | | | | |
| Extremely large VLDL | **0.59 [0.48,0.71]; P=1e-24** | -0.33 [-0.61,-0.05]; P=0.02 | 0.52 [0.34,0.71]; P=2e-08 | 1.5 [1.3,1.7]; P=7e-35 |
| Very large VLDL | **0.74 [0.63,0.85]; P=2e-37** | -0.26 [-0.54,0.01]; P=0.06 | 0.62 [0.44,0.80]; P=2e-11 | 1.8 [1.5,2.0]; P=2e-50 |
| Large VLDL | **0.80 [0.69,0.91]; P=4e-44** | -0.26 [-0.54,0.01]; P=0.06 | 0.71 [0.54,0.89]; P=5e-15 | 1.8 [1.5,2.0]; P=2e-49 |
| Medium VLDL | **0.73 [0.62,0.84]; P=6e-37** | -0.27 [-0.55,0.00]; P=0.05 | 0.67 [0.49,0.85]; P=3e-13 | 1.5 [1.3,1.7]; P=7e-36 |
| Small VLDL | **1.1 [1.0,1.2]; P=4e-87** | -0.21 [-0.47,0.06]; P=0.1 | 1.1 [1.0,1.3]; P=4e-38 | 2.0 [1.8,2.3]; P=3e-69 |
| Very small VLDL | **1.2 [1.1,1.3]; P=3e-97** | -0.31 [-0.57,-0.05]; P=0.02 | 1.2 [1.0,1.3]; P=3e-41 | 2.2 [2.0,2.4]; P=2e-83 |
| IDL | **0.99 [0.88,1.10]; P=3e-70** | -0.36 [-0.63,-0.09]; P=0.008 | 1.1 [0.9,1.2]; P=1e-32 | 1.8 [1.6,2.1]; P=6e-56 |
| Large LDL | **0.92 [0.81,1.03]; P=1e-59** | -0.40 [-0.67,-0.13]; P=0.004 | 0.99 [0.81,1.16]; P=3e-28 | 1.7 [1.5,2.0]; P=2e-49 |
| Medium LDL | **0.87 [0.76,0.98]; P=2e-53** | -0.41 [-0.68,-0.14]; P=0.003 | 0.95 [0.78,1.13]; P=3e-26 | 1.7 [1.5,1.9]; P=2e-46 |
| Small LDL | **0.86 [0.75,0.97]; P=2e-52** | -0.44 [-0.71,-0.17]; P=0.001 | 0.97 [0.80,1.15]; P=2e-27 | 1.7 [1.5,1.9]; P=7e-47 |
| Very large HDL | **1.2 [1.0,1.3]; P=4e-97** | 0.42 [0.16,0.69]; P=0.002 | 1.6 [1.4,1.7]; P=2e-70 | 1.4 [1.1,1.6]; P=5e-32 |
| Large HDL | **1.0 [0.9,1.1]; P=2e-73** | 0.42 [0.15,0.70]; P=0.002 | 1.3 [1.1,1.5]; P=3e-48 | 1.0 [0.8,1.3]; P=8e-18 |
| Medium HDL | 0.16 [0.05,0.28]; P=0.005 | -0.16 [-0.45,0.12]; P=0.3 | 0.36 [0.18,0.55]; P=0.0001 | -0.037 [-0.277,0.203]; P=0.8 |
| Small HDL | **0.46 [0.35,0.57]; P=1e-15** | -0.26 [-0.53,0.02]; P=0.07 | 0.53 [0.35,0.71]; P=1e-08 | 0.71 [0.47,0.94]; P=4e-09 |
| **Lipoprotein particle size** | | | | |
| VLDL particle size | **0.22 [0.10,0.33]; P=0.0002** | -0.19 [-0.48,0.09]; P=0.2 | 0.19 [0.01,0.38]; P=0.04 | 0.58 [0.34,0.82]; P=3e-06 |
| LDL particle size | -0.02 [-0.13,0.09]; P=0.7 | 0.35 [0.07,0.63]; P=0.02 | -0.18 [-0.37,0.00]; P=0.05 | -0.33 [-0.57,-0.09]; P=0.008 |
| HDL particle size | **0.85 [0.74,0.97]; P=4e-51** | 0.51 [0.23,0.78]; P=0.0003 | 1.2 [1.0,1.4]; P=6e-40 | 0.80 [0.57,1.04]; P=2e-11 |
| **Apolipoproteins** | | | | |
| Apolipoprotein B | **0.96 [0.85,1.07]; P=1e-64** | -0.47 [-0.73,-0.20]; P=0.0006 | 0.98 [0.81,1.15]; P=3e-28 | 1.9 [1.7,2.2]; P=1e-62 |
| Apolipoprotein A-I | **1.0 [0.9,1.1]; P=2e-73** | -0.0067 [-0.2765,0.2631]; P=1 | 1.3 [1.1,1.4]; P=3e-44 | 1.2 [1.0,1.5]; P=2e-26 |
| **Cholesterol** | | | | |
| Total C | **1.0 [0.9,1.1]; P=7e-76** | -0.36 [-0.62,-0.09]; P=0.009 | 1.2 [1.0,1.3]; P=7e-39 | 1.8 [1.5,2.0]; P=7e-52 |
| Non-HDL C | **0.80 [0.69,0.91]; P=2e-45** | -0.48 [-0.75,-0.21]; P=0.0005 | 0.86 [0.68,1.03]; P=3e-21 | 1.6 [1.4,1.9]; P=7e-44 |
| Remnant C | **0.91 [0.80,1.02]; P=2e-58** | -0.43 [-0.70,-0.16]; P=0.002 | 0.94 [0.76,1.12]; P=1e-25 | 1.8 [1.6,2.0]; P=4e-53 |
| VLDL C | **0.96 [0.85,1.07]; P=1e-64** | -0.34 [-0.60,-0.07]; P=0.01 | 0.97 [0.80,1.15]; P=2e-27 | 1.9 [1.6,2.1]; P=4e-57 |
| IDL C | **0.71 [0.60,0.83]; P=6e-36** | -0.46 [-0.73,-0.19]; P=0.001 | 0.77 [0.59,0.95]; P=3e-17 | 1.4 [1.2,1.7]; P=7e-33 |
| LDL C | **0.66 [0.55,0.77]; P=3e-31** | -0.49 [-0.77,-0.22]; P=0.0004 | 0.73 [0.55,0.91]; P=9e-16 | 1.4 [1.2,1.6]; P=6e-32 |
| HDL C | **0.85 [0.74,0.96]; P=4e-51** | 0.16 [-0.11,0.44]; P=0.2 | 1.2 [1.0,1.4]; P=2e-38 | 0.85 [0.61,1.08]; P=1e-12 |
| HDL2 C | **0.70 [0.59,0.81]; P=2e-34** | 0.20 [-0.08,0.48]; P=0.2 | 1.0 [0.8,1.2]; P=1e-28 | 0.57 [0.33,0.81]; P=2e-06 |
| HDL3 C | **1.4 [1.3,1.5]; P=1e-159** | -0.068 [-0.319,0.183]; P=0.6 | 1.8 [1.6,1.9]; P=2e-100 | 2.1 [1.9,2.3]; P=2e-81 |
| Esterified C | **1.0 [0.9,1.1]; P=1e-73** | -0.34 [-0.60,-0.07]; P=0.01 | 1.1 [1.0,1.3]; P=3e-38 | 1.7 [1.5,1.9]; P=9e-49 |
| Free C | **1.0 [0.9,1.1]; P=2e-76** | -0.39 [-0.66,-0.13]; P=0.003 | 1.2 [1.0,1.3]; P=2e-39 | 1.8 [1.6,2.1]; P=3e-56 |
| Esterified C (%) | -0.016 [-0.131,0.098]; P=0.8 | 0.19 [-0.10,0.47]; P=0.2 | 0.04 [-0.15,0.23]; P=0.7 | -0.31 [-0.55,-0.06]; P=0.01 |
| **Triglycerides** | | | | |
| Total TG | **1.4 [1.3,1.5]; P=9e-137** | 0.025 [-0.230,0.281]; P=0.8 | 1.4 [1.3,1.6]; P=2e-64 | 2.3 [2.1,2.5]; P=1e-92 |
| VLDL TG | **0.83 [0.72,0.95]; P=1e-48** | -0.19 [-0.46,0.09]; P=0.2 | 0.82 [0.64,1.00]; P=2e-19 | 1.6 [1.4,1.9]; P=3e-43 |
| IDL TG | **1.8 [1.7,1.9]; P=4e-266** | 0.25 [0.02,0.49]; P=0.04 | 2.0 [1.8,2.1]; P=5e-141 | 2.9 [2.7,3.1]; P=8e-167 |
| LDL TG | **1.8 [1.7,1.9]; P=6e-280** | 0.33 [0.10,0.57]; P=0.005 | 2.1 [1.9,2.2]; P=2e-157 | 2.8 [2.6,3.0]; P=5e-161 |
| HDL TG | **1.8 [1.7,1.9]; P=1e-276** | 0.56 [0.32,0.80]; P=4e-06 | 2.1 [1.9,2.2]; P=7e-152 | 2.5 [2.3,2.7]; P=3e-121 |
| **Phospholipids** | | | | |
| Total PL | **1.4 [1.3,1.5]; P=3e-139** | -0.087 [-0.345,0.170]; P=0.5 | 1.5 [1.4,1.7]; P=7e-71 | 2.0 [1.8,2.2]; P=1e-69 |
| VLDL PL | **1.1 [1.0,1.2]; P=2e-83** | -0.29 [-0.56,-0.03]; P=0.03 | 1.1 [0.9,1.3]; P=3e-36 | 2.1 [1.8,2.3]; P=9e-72 |
| IDL PL | **0.99 [0.88,1.10]; P=1e-69** | -0.35 [-0.62,-0.08]; P=0.01 | 1.0 [0.9,1.2]; P=5e-32 | 1.8 [1.6,2.0]; P=3e-54 |
| LDL PL | **0.91 [0.80,1.02]; P=4e-59** | -0.43 [-0.70,-0.16]; P=0.002 | 1.00 [0.82,1.17]; P=6e-29 | 1.7 [1.5,2.0]; P=4e-49 |
| HDL PL | **0.89 [0.78,1.00]; P=2e-55** | 0.25 [-0.02,0.53]; P=0.07 | 1.2 [1.1,1.4]; P=3e-41 | 0.81 [0.58,1.05]; P=1e-11 |
| Cholines | **1.4 [1.3,1.5]; P=8e-158** | -0.00 [-0.25,0.25]; P=1 | 1.7 [1.5,1.8]; P=1e-85 | 2.1 [1.9,2.3]; P=7e-81 |
| Sphingomyelin | **1.3 [1.2,1.4]; P=2e-124** | -0.027 [-0.285,0.232]; P=0.8 | 1.5 [1.3,1.7]; P=5e-67 | 1.9 [1.7,2.1]; P=1e-63 |
| Phosphoglycerides | **1.3 [1.2,1.4]; P=7e-129** | -0.10 [-0.36,0.15]; P=0.4 | 1.5 [1.4,1.7]; P=2e-71 | 2.0 [1.8,2.3]; P=9e-71 |
| **Fatty acids** | | | | |
| Total FA | **1.4 [1.3,1.5]; P=5e-156** | -0.092 [-0.344,0.159]; P=0.5 | 1.6 [1.5,1.8]; P=4e-83 | 2.3 [2.1,2.5]; P=1e-94 |
| Saturated FA | **1.5 [1.4,1.6]; P=6e-167** | -0.059 [-0.309,0.191]; P=0.6 | 1.7 [1.5,1.8]; P=2e-89 | 2.4 [2.2,2.6]; P=2e-103 |
| MUFA | **1.4 [1.3,1.5]; P=1e-150** | -0.054 [-0.306,0.198]; P=0.7 | 1.6 [1.4,1.7]; P=3e-78 | 2.3 [2.1,2.5]; P=1e-94 |
| PUFA | **1.2 [1.1,1.3]; P=4e-102** | -0.14 [-0.40,0.12]; P=0.3 | 1.3 [1.2,1.5]; P=3e-54 | 1.9 [1.7,2.1]; P=4e-62 |
| Omega-6 FA | **1.1 [1.0,1.2]; P=5e-91** | -0.22 [-0.48,0.05]; P=0.1 | 1.3 [1.1,1.4]; P=3e-48 | 1.9 [1.6,2.1]; P=1e-59 |
| Linoleic acid | **0.97 [0.86,1.08]; P=2e-67** | -0.32 [-0.59,-0.06]; P=0.02 | 1.1 [0.9,1.3]; P=1e-35 | 1.7 [1.5,1.9]; P=1e-49 |
| Omega-3 FA | **1.1 [1.0,1.2]; P=9e-96** | 0.29 [0.02,0.55]; P=0.03 | 1.4 [1.2,1.6]; P=2e-55 | 1.6 [1.3,1.8]; P=3e-41 |
| Docosahexaenoic acid | **1.3 [1.2,1.4]; P=1e-131** | 0.40 [0.14,0.66]; P=0.002 | 1.6 [1.4,1.7]; P=1e-72 | 1.8 [1.6,2.0]; P=6e-58 |
| **Fatty acid ratios** | | | | |
| Saturated FA (%) | **0.64 [0.53,0.75]; P=1e-28** | 0.15 [-0.13,0.43]; P=0.3 | 0.64 [0.45,0.82]; P=1e-11 | 1.0 [0.8,1.3]; P=1e-16 |
| MUFA (%) | **0.76 [0.64,0.87]; P=8e-40** | 0.053 [-0.223,0.329]; P=0.7 | 0.78 [0.60,0.96]; P=4e-17 | 1.3 [1.0,1.5]; P=2e-26 |
| PUFA (%) | **-1.1 [-1.2,-0.9]; P=2e-78** | -0.11 [-0.38,0.15]; P=0.4 | -1.0 [-1.2,-0.9]; P=9e-32 | -1.8 [-2.1,-1.6]; P=5e-55 |
| Omega-6 FA (%) | **-1.2 [-1.3,-1.1]; P=3e-96** | -0.27 [-0.54,-0.01]; P=0.04 | -1.2 [-1.4,-1.0]; P=2e-41 | -1.9 [-2.1,-1.6]; P=1e-57 |
| Linoleic acid (%) | **-1.2 [-1.3,-1.1]; P=1e-96** | -0.43 [-0.70,-0.17]; P=0.001 | -1.2 [-1.4,-1.1]; P=1e-43 | -1.7 [-1.9,-1.5]; P=8e-48 |
| Omega-3 FA (%) | 0.024 [-0.089,0.138]; P=0.7 | 0.52 [0.24,0.80]; P=0.0003 | 0.17 [-0.01,0.35]; P=0.07 | -0.47 [-0.71,-0.23]; P=0.0002 |
| Docosahexaenoic acid (%) | **0.53 [0.42,0.65]; P=2e-20** | 0.69 [0.41,0.97]; P=1e-06 | 0.74 [0.56,0.92]; P=3e-15 | 0.35 [0.11,0.59]; P=0.004 |
| Degree of unsaturation | **-0.67 [-0.78,-0.56]; P=1e-31** | 0.19 [-0.08,0.46]; P=0.2 | -0.61 [-0.79,-0.43]; P=2e-11 | -1.5 [-1.7,-1.3]; P=1e-35 |
| **Amino acids** | | | | |
| Alanine | **0.45 [0.33,0.56]; P=1e-14** | -0.35 [-0.63,-0.07]; P=0.01 | 0.43 [0.25,0.62]; P=4e-06 | 0.93 [0.69,1.17]; P=3e-14 |
| Glutamine | **-1.1 [-1.2,-1.0]; P=3e-85** | -1.1 [-1.4,-0.9]; P=7e-17 | -1.1 [-1.3,-0.9]; P=5e-37 | -1.1 [-1.3,-0.8]; P=4e-20 |
| Glycine | **-0.87 [-0.98,-0.76]; P=2e-53** | -0.92 [-1.19,-0.64]; P=5e-11 | -1.0 [-1.2,-0.8]; P=3e-29 | -0.85 [-1.09,-0.62]; P=9e-13 |
| *Branched-chain amino acids* | | | | |
| Isoleucine | 0.075 [-0.038,0.189]; P=0.2 | -0.23 [-0.51,0.06]; P=0.1 | 0.03 [-0.16,0.21]; P=0.8 | 0.32 [0.08,0.56]; P=0.009 |
| Leucine | -0.051 [-0.164,0.062]; P=0.4 | -0.31 [-0.59,-0.03]; P=0.03 | -0.14 [-0.33,0.04]; P=0.1 | 0.03 [-0.21,0.27]; P=0.8 |
| Valine | **-0.66 [-0.77,-0.55]; P=3e-31** | -0.38 [-0.66,-0.11]; P=0.007 | -0.80 [-0.98,-0.62]; P=2e-18 | -1.1 [-1.3,-0.9]; P=4e-20 |
| *Aromatic amino acids* | | | | |
| Phenylalanine | **1.3 [1.1,1.4]; P=2e-116** | 0.44 [0.17,0.70]; P=0.001 | 1.3 [1.1,1.5]; P=5e-49 | 1.7 [1.4,1.9]; P=3e-48 |
| Tyrosine | **-0.78 [-0.89,-0.67]; P=7e-43** | -0.68 [-0.95,-0.40]; P=2e-06 | -0.93 [-1.11,-0.75]; P=2e-24 | -0.91 [-1.14,-0.67]; P=3e-14 |
| Histidine | **0.69 [0.58,0.80]; P=2e-34** | 0.11 [-0.16,0.38]; P=0.4 | 0.81 [0.64,0.99]; P=3e-19 | 0.97 [0.74,1.20]; P=2e-16 |
| **Glycolysis and gluconeogenesis** | | | | |
| Glucose | **-0.59 [-0.70,-0.48]; P=8e-26** | -0.34 [-0.61,-0.06]; P=0.02 | -0.57 [-0.75,-0.39]; P=5e-10 | -0.70 [-0.93,-0.47]; P=4e-09 |
| Lactate | **0.28 [0.17,0.39]; P=1e-06** | -0.031 [-0.312,0.250]; P=0.8 | 0.29 [0.11,0.47]; P=0.002 | 0.42 [0.18,0.66]; P=0.0006 |
| Pyruvate | **0.37 [0.26,0.48]; P=2e-10** | 0.062 [-0.221,0.346]; P=0.7 | 0.36 [0.17,0.54]; P=0.0002 | 0.62 [0.38,0.86]; P=5e-07 |
| Citrate | 0.036 [-0.077,0.149]; P=0.5 | -0.22 [-0.50,0.06]; P=0.1 | -0.025 [-0.209,0.158]; P=0.8 | 0.60 [0.36,0.84]; P=7e-07 |
| Glycerol | **-0.24 [-0.36,-0.13]; P=6e-05** | -0.41 [-0.71,-0.12]; P=0.006 | -0.41 [-0.60,-0.22]; P=2e-05 | -0.42 [-0.68,-0.16]; P=0.001 |
| **Ketone bodies** | | | | |
| Acetoacetate | **-0.27 [-0.38,-0.16]; P=3e-06** | -0.40 [-0.68,-0.12]; P=0.005 | -0.33 [-0.51,-0.14]; P=0.0005 | -0.21 [-0.45,0.03]; P=0.09 |
| Beta-hydroxybutyrate | 0.077 [-0.037,0.191]; P=0.2 | -0.17 [-0.46,0.12]; P=0.2 | 0.057 [-0.127,0.242]; P=0.5 | 0.32 [0.08,0.57]; P=0.009 |
| **Miscellaneous** | | | | |
| Creatinine | **-0.99 [-1.10,-0.88]; P=1e-70** | -1.1 [-1.3,-0.8]; P=2e-14 | -1.0 [-1.2,-0.8]; P=1e-28 | -0.77 [-1.01,-0.54]; P=5e-11 |
| Albumin | **-0.88 [-0.99,-0.77]; P=1e-56** | -0.37 [-0.64,-0.10]; P=0.007 | -0.81 [-0.99,-0.63]; P=2e-19 | -1.2 [-1.5,-1.0]; P=8e-26 |
| Acetate | -0.012 [-0.126,0.102]; P=0.8 | -0.45 [-0.73,-0.17]; P=0.002 | 0.10 [-0.08,0.29]; P=0.3 | 0.17 [-0.07,0.41]; P=0.2 |
| **Inflammation markers** | | | | |
| Glycoprotein acetyls | **0.99 [0.88,1.10]; P=5e-71** | 0.10 [-0.17,0.37]; P=0.5 | 0.98 [0.80,1.15]; P=9e-28 | 1.4 [1.2,1.7]; P=2e-34 |
| C-reactive protein | **1.1 [1.0,1.2]; P=1e-86** | 0.69 [0.42,0.96]; P=5e-07 | 1.3 [1.1,1.5]; P=5e-47 | 1.1 [0.8,1.3]; P=3e-19 |
| **Hormone related** | | | | |
| Insulin | 0.19 [0.07,0.30]; P=0.002 | 0.0048 [-0.2998,0.3094]; P=1 | 0.085 [-0.108,0.277]; P=0.4 | 0.59 [0.34,0.84]; P=3e-06 |
| Leptin | **0.67 [0.50,0.85]; P=6e-14** | 0.61 [-0.07,1.30]; P=0.08 | 0.83 [0.49,1.17]; P=2e-06 | 0.82 [0.34,1.29]; P=0.0007 |
| Adiponectin | -0.038 [-0.219,0.142]; P=0.7 | 0.36 [-0.34,1.06]; P=0.3 | 0.069 [-0.282,0.420]; P=0.7 | -0.24 [-0.72,0.24]; P=0.3 |
| Vitamin D | -0.21 [-0.34,-0.09]; P=0.0009 | 0.021 [-0.297,0.339]; P=0.9 | -0.20 [-0.43,0.02]; P=0.07 | 0.10 [-0.17,0.38]; P=0.5 |
| SHBG | **2.5 [2.4,2.6]; P=0e+00** | 1.6 [1.4,1.8]; P=6e-55 | 2.8 [2.7,3.0]; P=0e+00 | 3.2 [3.0,3.4]; P=4e-281 |
| Testosterone | **1.4 [1.3,1.5]; P=1e-152** | 1.6 [1.4,1.9]; P=1e-36 | 1.4 [1.2,1.6]; P=1e-61 | 1.7 [1.5,2.0]; P=3e-52 |

The cross-sectional associations between pregnancy and 87 metabolic measures were meta-analysed for 4260 women across three independent population-based cohorts. The association magnitudes were defined as the metabolic difference between pregnant (n=322) and non-pregnant women (n=3938). The cross-sectional associations with the trimesters were meta-analysed for NFBC1966 and YFS (at 2001 survey). The related association magnitudes were defined as the metabolic differences between the first (n=48), the second (n=116) and the third trimesters (n=67) and the non-pregnant women (n=2588). The associations were adjusted for age. To ease the comparison across the metabolic measures, the associations were reported in SD units. In the cross-sectional analysis, 75 out of 87 metabolic measures were significant after the multiple testing comparison, either using modified-Bonferroni correction (P < 0.05/61, 61 is the number of PCs explaining over 99% of variation in the data) or conventional Bonferroni correction (P < 0.05/124, 124 is the total number of metabolic measures in the present study). The significant associations in the meta-analysis (column 2) are indicated in bold.

Abbreviations: VLDL, very-low-density lipoprotein; IDL, intermediate-density lipoprotein; LDL, low-density lipoprotein; HDL, high-density lipoprotein; C, cholesterol; PL, phospholipids; PUFA, polyunsaturated fatty acids; MUFA, monounsaturated fatty acids; SHBG, sex hormone-binding globulin; cu, standardised concentration unit.

**Table S3. Cross-sectional metabolic differences associated with pregnancy and pregnancy trimesters in absolute concentration units.**

| **Metabolic measures** | **Mean (SD)** | **Absolute**  **difference associated with pregnancy**  **Beta [95%CI]** | **Absolute**  **difference**  **associated with**  **the first trimester**  **Beta [95%CI]** | **Absolute**  **difference**  **associated with the second trimester**  **Beta [95%CI]** | **Absolute**  **difference**  **associated with**  **the third trimester**  **Beta [95%CI]** |
| --- | --- | --- | --- | --- | --- |
| **Lipoprotein subclass total lipids** | | | | | |
| Extremely large VLDL (µmol/L) | 20 (19) | 11 [8, 13] | -5.8 [-10.7, -0.9] | 9.4 [6.1, 12.6] | 27 [22, 31] |
| Very large VLDL (µmol/L) | 37 (46) | 31 [27, 36] | -12 [-24, 1] | 27 [19, 36] | 83 [72, 94] |
| Large VLDL (µmol/L) | 139 (154) | 116 [100, 133] | -43 [-88, 2] | 113 [83, 143] | 295 [256, 333] |
| Medium VLDL (mmol/L) | 0.40 (0.25) | 0.20 [0.17, 0.23] | -0.077 [-0.150, -0.004] | 0.18 [0.13, 0.23] | 0.51 [0.45, 0.57] |
| Small VLDL (mmol/L) | 0.54 (0.24) | 0.33 [0.30, 0.36] | -0.053 [-0.116, 0.009] | 0.31 [0.27, 0.35] | 0.66 [0.61, 0.71] |
| Very small VLDL (mmol/L) | 0.58 (0.18) | 0.24 [0.22, 0.26] | -0.051 [-0.096, -0.005] | 0.22 [0.20, 0.25] | 0.48 [0.45, 0.52] |
| IDL (mmol/L) | 1.3 (0.4) | 0.44 [0.40, 0.48] | -0.13 [-0.24, -0.03] | 0.44 [0.38, 0.51] | 0.85 [0.76, 0.93] |
| Large LDL (mmol/L) | 1.4 (0.4) | 0.48 [0.43, 0.53] | -0.18 [-0.30, -0.05] | 0.50 [0.42, 0.58] | 0.96 [0.85, 1.06] |
| Medium LDL (mmol/L) | 0.79 (0.26) | 0.28 [0.25, 0.31] | -0.11 [-0.19, -0.04] | 0.30 [0.25, 0.35] | 0.57 [0.51, 0.64] |
| Small LDL (mmol/L) | 0.50 (0.16) | 0.16 [0.15, 0.18] | -0.072 [-0.117, -0.027] | 0.18 [0.15, 0.21] | 0.34 [0.30, 0.38] |
| Very large HDL (mmol/L) | 0.66 (0.26) | 0.33 [0.30, 0.36] | 0.11 [0.04, 0.18] | 0.46 [0.41, 0.50] | 0.40 [0.34, 0.46] |
| Large HDL (mmol/L) | 1.0 (0.4) | 0.48 [0.44, 0.53] | 0.18 [0.06, 0.29] | 0.66 [0.59, 0.74] | 0.52 [0.42, 0.62] |
| Medium HDL (mmol/L) | 0.93 (0.23) | 0.042  [0.015, 0.070] | -0.048  [-0.124, 0.029] | 0.099  [0.050, 0.148] | -0.0022  [-0.0664, 0.0620] |
| Small HDL (mmol/L) | 1.1 (0.2) | 0.085 [0.066, 0.104] | -0.049 [-0.103, 0.006] | 0.11 [0.07, 0.14] | 0.14 [0.09, 0.18] |
| **Lipoprotein particle size** | | | | | |
| VLDL particle size (nm) | 35 (1) | 0.22 [0.10, 0.33] | -0.21 [-0.51, 0.10] | 0.20 [0.00, 0.40] | 0.62 [0.36, 0.88] |
| LDL particle size (nm) | 24 (0) | -0.0042  [-0.0212, 0.0127] | 0.051  [0.010, 0.091] | -0.026  [-0.053, 0.000] | -0.047  [-0.082, -0.013] |
| HDL particle size (nm) | 10 (0) | 0.21 [0.19, 0.24] | 0.13 [0.06, 0.20] | 0.31 [0.26, 0.35] | 0.20 [0.14, 0.26] |
| **Apolipoproteins** | | | | | |
| Apolipoprotein B (g/L) | 0.91 (0.23) | 0.26 [0.24, 0.29] | -0.11 [-0.17, -0.04] | 0.26 [0.22, 0.30] | 0.56 [0.50, 0.61] |
| Apolipoprotein A-I (g/L) | 1.7 (0.3) | 0.30  [0.27, 0.33] | -0.0078  [-0.0948, 0.0792] | 0.41  [0.35, 0.46] | 0.42  [0.34, 0.49] |
| **Cholesterol** | | | | | |
| Total C (mmol/L) | 5.1 (1.2) | 1.4 [1.3, 1.6] | -0.46 [-0.81, -0.11] | 1.6 [1.4, 1.8] | 2.6 [2.3, 2.9] |
| Non-HDL C (mmol/L) | 3.4 (1.1) | 1.0 [0.9, 1.2] | -0.52 [-0.82, -0.21] | 1.0 [0.9, 1.2] | 2.2 [1.9, 2.5] |
| Remnant C (mmol/L) | 1.6 (0.5) | 0.54 [0.48, 0.59] | -0.21 [-0.35, -0.07] | 0.52 [0.43, 0.61] | 1.1 [1.0, 1.2] |
| VLDL C (mmol/L) | 0.75 (0.30) | 0.34 [0.30, 0.37] | -0.098 [-0.176, -0.020] | 0.31 [0.26, 0.36] | 0.70 [0.63, 0.77] |
| IDL C (mmol/L) | 0.83 (0.23) | 0.20 [0.17, 0.23] | -0.11 [-0.18, -0.04] | 0.21 [0.16, 0.25] | 0.42 [0.36, 0.47] |
| LDL C (mmol/L) | 1.8 (0.6) | 0.49 [0.43, 0.56] | -0.31 [-0.49, -0.13] | 0.53 [0.42, 0.65] | 1.1 [0.9, 1.2] |
| HDL C (mmol/L) | 1.7 (0.4) | 0.38 [0.34, 0.43] | 0.058 [-0.066, 0.181] | 0.57 [0.49, 0.65] | 0.43 [0.33, 0.53] |
| HDL_2_ C (mmol/L) | 1.2 (0.4) | 0.30 [0.26, 0.34] | 0.062 [-0.051, 0.176] | 0.46 [0.39, 0.54] | 0.29 [0.19, 0.38] |
| HDL_3_ C (mmol/L) | 0.54 (0.05) | 0.082  [0.076, 0.087] | -0.0038  [-0.0184, 0.0108] | 0.11  [0.10, 0.11] | 0.13  [0.12, 0.14] |
| Esterified C (mmol/L) | 3.6 (0.9) | 1.0 [0.9, 1.1] | -0.32 [-0.57, -0.06] | 1.2 [1.0, 1.3] | 1.8 [1.6, 2.0] |
| Free C (mmol/L) | 1.5 (0.3) | 0.41 [0.37, 0.45] | -0.14 [-0.24, -0.04] | 0.46 [0.40, 0.52] | 0.77 [0.69, 0.86] |
| Esterified C (%) | 71 (1) | -0.035 [-0.178, 0.109] | 0.21 [-0.13, 0.56] | 0.033 [-0.197, 0.263] | -0.40 [-0.70, -0.10] |

| **Triglycerides** | | | | | |
| --- | --- | --- | --- | --- | --- |
| Total TG (mmol/L) | 1.0 (0.6) | 0.89 [0.83, 0.95] | -0.019 [-0.160, 0.122] | 0.89 [0.80, 0.98] | 1.7 [1.6, 1.8] |
| VLDL TG (mmol/L) | 0.56 (0.38) | 0.37 [0.33, 0.41] | -0.092 [-0.199, 0.015] | 0.34 [0.27, 0.41] | 0.87 [0.78, 0.96] |
| IDL TG (mmol/L) | 0.12 (0.06) | 0.12 [0.12, 0.13] | 0.011 [-0.001, 0.023] | 0.13 [0.12, 0.13] | 0.21 [0.20, 0.22] |
| LDL TG (mmol/L) | 0.18 (0.11) | 0.24 [0.23, 0.25] | 0.026 [0.005, 0.047] | 0.25 [0.23, 0.26] | 0.39 [0.37, 0.41] |
| HDL TG (mmol/L) | 0.15 (0.07) | 0.15 [0.15, 0.16] | 0.036 [0.020, 0.051] | 0.17 [0.16, 0.18] | 0.22 [0.20, 0.23] |
| **Phospholipids** | | | | | |
| Total PL (mmol/L) | 3.3 (0.6) | 0.92 [0.86, 0.98] | -0.065 [-0.242, 0.112] | 1.1 [1.0, 1.2] | 1.5 [1.3, 1.6] |
| VLDL PL (mmol/L) | 0.41 (0.18) | 0.23 [0.21, 0.25] | -0.052 [-0.099, -0.005] | 0.22 [0.19, 0.25] | 0.48 [0.44, 0.52] |
| IDL PL (mmol/L) | 0.34 (0.09) | 0.11 [0.10, 0.12] | -0.033 [-0.059, -0.007] | 0.11 [0.09, 0.13] | 0.21 [0.18, 0.23] |
| LDL PL (mmol/L) | 0.73 (0.17) | 0.18 [0.16, 0.20] | -0.078 [-0.128, -0.028] | 0.20 [0.17, 0.23] | 0.36 [0.32, 0.41] |
| HDL PL (mmol/L) | 1.8 (0.4) | 0.39 [0.34, 0.43] | 0.098 [-0.022, 0.219] | 0.57 [0.50, 0.65] | 0.39 [0.29, 0.49] |
| Cholines (mmol/L) | 2.2 (0.5) | 0.77 [0.72, 0.82] | -0.012 [-0.143, 0.119] | 0.92 [0.84, 1.01] | 1.2 [1.1, 1.3] |
| Sphingomyelin (mmol/L) | 0.56 (0.10) | 0.15 [0.14, 0.16] | -0.006 [-0.036, 0.024] | 0.18 [0.16, 0.20] | 0.24 [0.22, 0.27] |
| Phosphoglycerides (mmol/L) | 2.2 (0.5) | 0.76 [0.71, 0.82] | -0.066 [-0.206, 0.074] | 0.91 [0.82, 1.00] | 1.3 [1.1, 1.4] |
| **Fatty acids** | | | | | |
| Total FA (mmol/L) | 12 (3) | 4.8 [4.5, 5.2] | -0.33 [-1.12, 0.45] | 5.6 [5.1, 6.1] | 8.3 [7.6, 9.0] |
| Saturated FA (mmol/L) | 4.7 (1.2) | 2.1 [1.9, 2.2] | -0.088 [-0.400, 0.223] | 2.3 [2.1, 2.5] | 3.5 [3.3, 3.8] |
| MUFA (mmol/L) | 3.1 (1.0) | 1.6 [1.4, 1.7] | -0.067 [-0.314, 0.180] | 1.7 [1.6, 1.9] | 2.7 [2.5, 2.9] |
| PUFA (mmol/L) | 4.6 (1.0) | 1.3 [1.2, 1.4] | -0.17 [-0.45, 0.11] | 1.5 [1.3, 1.7] | 2.2 [2.0, 2.5] |
| Omega-6 FA (mmol/L) | 4.1 (0.8) | 1.1 [1.0, 1.2] | -0.21 [-0.46, 0.03] | 1.2 [1.1, 1.4] | 1.9 [1.7, 2.1] |
| Linoleic acid (mmol/L) | 3.3 (0.7) | 0.77 [0.69, 0.84] | -0.25 [-0.46, -0.05] | 0.87 [0.74, 1.00] | 1.5 [1.3, 1.6] |
| Omega-3 FA (mmol/L) | 0.55 (0.17) | 0.20 [0.19, 0.22] | 0.038 [-0.008, 0.083] | 0.26 [0.23, 0.29] | 0.30 [0.26, 0.34] |
| Docosahexaenoic acid (mmol/L) | 0.19 (0.07) | 0.099 [0.092, 0.106] | 0.022 [0.005, 0.040] | 0.12 [0.11, 0.14] | 0.15 [0.14, 0.17] |
| **Fatty acid ratios** | | | | | |
| Saturated FA (%) | 38 (2) | 1.2 [1.0, 1.4] | 0.27 [-0.23, 0.78] | 1.2 [0.8, 1.5] | 1.9 [1.5, 2.3] |
| MUFA (%) | 24 (3) | 2.0 [1.7, 2.3] | 0.12 [-0.62, 0.85] | 2.1 [1.6, 2.6] | 3.6 [3.0, 4.2] |
| PUFA (%) | 38 (3) | -3.2 [-3.6, -2.9] | -0.39 [-1.22, 0.45] | -3.3 [-3.8, -2.7] | -5.5 [-6.2, -4.8] |
| Omega-6 FA (%) | 33 (3) | -3.2 [-3.6, -2.9] | -0.83 [-1.59, -0.07] | -3.4 [-3.9, -2.9] | -5.1 [-5.8, -4.5] |
| Linoleic acid (%) | 27 (3) | -3.2 [-3.5, -2.9] | -1.3 [-2.0, -0.5] | -3.4 [-3.9, -2.9] | -4.6 [-5.2, -3.9] |
| Omega-3 FA (%) | 4.4 (1.0) | 0.04 [-0.07, 0.15] | 0.47 [0.22, 0.73] | 0.14 [-0.03, 0.31] | -0.37 [-0.59, -0.15] |
| Docosahexaenoic acid (%) | 1.5 (0.4) | 0.18 [0.14, 0.22] | 0.22 [0.13, 0.32] | 0.26 [0.19, 0.32] | 0.10 [0.02, 0.19] |
| Degree of unsaturation | 1.2 (0.1) | -0.035 [-0.041, -0.029] | 0.01 [0.00, 0.02] | -0.032 [-0.042, -0.023] | -0.077 [-0.089, -0.064] |
| **Amino acids** | | | | | |
| Alanine (µmol/L) | 425 (70) | 32 [24, 40] | -29 [-52, -6] | 32 [17, 46] | 74 [55, 93] |
| Glutamine (µmol/L) | 512 (85) | -86 [-95, -76] | -103 [-130, -76] | -97 [-114, -80] | -95 [-117, -73] |
| Glycine (µmol/L) | 343 (77) | -60 [-69, -52] | -67 [-90, -45] | -75 [-89, -60] | -63 [-82, -43] |
| *Branched-chain amino acids* | | | | | |
| Isoleucine (µmol/L) | 50 (14) | 0.94 [-0.65, 2.52] | -3.3 [-7.1, 0.6] | 0.42 [-2.10, 2.93] | 5.0 [1.7, 8.3] |
| Leucine (µmol/L) | 80 (17) | -1.1 [-3.1, 0.8] | -5.4 [-10.2, -0.5] | -2.4 [-5.6, 0.7] | 0.085 [-4.021, 4.191] |
| Valine (µmol/L) | 197 (43) | -26 [-31, -21] | -17 [-29, -5] | -32 [-40, -24] | -42 [-52, -32] |
| *Aromatic amino acids* | | | | | |
| Phenylalanine (µmol/L) | 84 (14) | 19 [18, 21] | 6.3 [2.3, 10.3] | 19 [17, 22] | 26 [23, 30] |
| Tyrosine (µmol/L) | 53 (13) | -9.2 [-10.7, -7.8] | -8.2 [-11.8, -4.5] | -11 [-13, -9] | -10 [-13, -7] |
| Histidine (µmol/L) | 70 (12) | 9.1 [7.7, 10.5] | 1.2 [-2.5, 4.9] | 11 [9, 14] | 13 [10, 16] |
| **Glycolysis and gluconeogenesis** | | | | | |
| Glucose (mmol/L) | 4.6 (0.6) | -0.35 [-0.43, -0.28] | -0.25 [-0.47, -0.03] | -0.38 [-0.52, -0.24] | -0.47 [-0.65, -0.28] |
| Lactate (mmol/L) | 1.4 (0.4) | 0.11 [0.07, 0.15] | -0.025 [-0.152, 0.103] | 0.15 [0.07, 0.23] | 0.19 [0.09, 0.30] |
| Pyruvate (µmol/L) | 85 (26) | 9.0 [6.2, 11.9] | -0.012 [-8.097, 8.073] | 11 [5, 16] | 19 [12, 26] |
| Citrate (µmol/L) | 112 (20) | 0.50 [-1.79, 2.79] | -5.1 [-10.9, 0.7] | -0.94 [-4.78, 2.89] | 13 [8, 18] |
| Glycerol (µmol/L) | 103 (42) | -8.4 [-13.1, -3.8] | -12 [-23, -1] | -13 [-20, -6] | -13 [-23, -4] |
| **Ketone bodies** | | | | | |
| Acetoacetate (µmol/L) | 61 (41) | -9.0 [-13.4, -4.5] | -9.5 [-20.1, 1.1] | -11 [-18, -4] | -9.1 [-18.0, -0.2] |
| Beta-hydroxybutyrate (µmol/L) | 183 (107) | 0.55 [-10.19, 11.29] | -14 [-40, 13] | -3.3 [-18.8, 12.2] | 14 [-6, 35] |
| **Miscellaneous** | | | | | |
| Creatinine (µmol/L) | 56 (10) | -9.5 [-10.7, -8.4] | -11 [-14, -8] | -9.8 [-11.7, -7.8] | -7.8 [-10.4, -5.2] |
| Albumin (cu) | 0.10 (0.01) | -0.009 [-0.010, -0.008] | -0.0061 [-0.0107, -0.0016] | -0.011 [-0.014, -0.008] | -0.017 [-0.021, -0.013] |
| Acetate (µmol/L) | 46 (13) | -0.13 [-1.49, 1.24] | -4.7 [-7.9, -1.5] | 1.3 [-0.8, 3.5] | 1.8 [-1.0, 4.5] |
| **Inflammation markers** | | | | | |
| Glycoprotein acetyls (mmol/L) | 1.3 (0.2) | 0.26 [0.23, 0.29] | 0.02 [-0.05, 0.09] | 0.27 [0.22, 0.31] | 0.40 [0.34, 0.46] |
| C-reactive protein (mg/L) | 1.7 (2.9) | 2.6 [2.2, 2.9] | 1.2 [0.3, 2.1] | 3.1 [2.5, 3.6] | 2.5 [1.8, 3.3] |
| **Hormone related** | | | | | |
| Insulin (IU/L) | 6.7 (3.8) | 0.52 [0.13, 0.91] | -0.22 [-1.20, 0.76] | 0.24 [-0.40, 0.89] | 1.7 [0.9, 2.5] |
| Leptin (ng/mL) | 15 (11) | 7.5 [5.5, 9.4] | 5.0 [-2.0, 12.1] | 8.9 [5.4, 12.5] | 9.0 [4.2, 13.9] |
| Adiponectin (ug/mL) | 9.2 (4.3) | 0.059 [-0.713, 0.832] | 1.6 [-1.4, 4.6] | 0.53 [-0.98, 2.04] | -0.77 [-2.85, 1.31] |
| Vitamin D (cu) | 23 (10) | -1.6 [-2.5, -0.6] | -0.76 [-3.52, 2.00] | -1.7 [-3.7, 0.3] | 2.8 [0.4, 5.3] |
| SHBG (nmol/L) | 84 (95) | 292 [284, 299] | 135 [121, 148] | 317 [308, 325] | 422 [411, 433] |
| Testosterone (nmol/L) | 1.4 (0.7) | 1.2 [1.1, 1.2] | 1.4 [1.2, 1.5] | 1.3 [1.2, 1.4] | 1.6 [1.4, 1.7] |

Means and SDs are the average of the means and SDs in each cohort. The absolute metabolic differences (e.g. in mmol/L) between pregnant and non-pregnant women are reported. Similarly, the absolute metabolic differences between pregnant women at the three trimesters and non-pregnant women are also denoted. Associations reported in absolute concentration units facilitate the biological interpretations. The metabolic associations were adjusted for age. The study design and the abbreviations are as in Table S2.

**Table S4. Cross-sectional metabolic differences associated with pregnancy and pregnancy trimesters in relative to non-pregnant women.**

| **Metabolic measures** | **Relative**  **difference (%) associated with pregnancy**  **Beta [95%CI]** | **Relative**  **difference (%) associated with the**  **first trimester**  **Beta [95%CI]** | **Relative**  **difference (%) associated with the second trimester**  **Beta [95%CI]** | **Relative**  **difference (%) associated with the**  **third trimester**  **Beta [95%CI]** |
| --- | --- | --- | --- | --- |
| **Lipoprotein subclass total lipids** | | | | |
| Extremely large VLDL | 54 [43, 64] | -31 [-56, -5] | 50 [32, 67] | 141 [119, 163] |
| Very large VLDL | 94 [79, 108] | -33 [-68, 2] | 76 [53, 99] | 229 [199, 259] |
| Large VLDL | 97 [83, 110] | -32 [-65, 2] | 83 [61, 104] | 212 [184, 240] |
| Medium VLDL | 54 [46, 61] | -19 [-37, -1] | 44 [33, 56] | 124 [108, 139] |
| Small VLDL | 63 [58, 68] | -10 [-22, 2] | 59 [51, 67] | 126 [115, 136] |
| Very small VLDL | 43 [39, 46] | -8.8 [-16.7, -0.9] | 40 [34, 45] | 85 [78, 92] |
| IDL | 34 [31, 37] | -10 [-18, -2] | 34 [29, 39] | 65 [59, 72] |
| Large LDL | 34 [31, 37] | -12 [-21, -4] | 34 [29, 40] | 66 [59, 73] |
| Medium LDL | 36 [32, 40] | -14 [-24, -5] | 37 [31, 44] | 72 [64, 80] |
| Small LDL | 33 [30, 37] | -15 [-24, -5] | 36 [30, 42] | 68 [60, 75] |
| Very large HDL | 50 [45, 54] | 19 [7, 30] | 80 [72, 88] | 68 [58, 78] |
| Large HDL | 48 [44, 53] | 18 [6, 30] | 68 [60, 75] | 53 [43, 63] |
| Medium HDL | 4.6 [1.7, 7.6] | -4.8 [-12.6, 2.9] | 10 [5, 15] | -0.20 [-6.60, 6.21] |
| Small HDL | 8.0 [6.2, 9.8] | -4.3 [-9.2, 0.5] | 9.7 [6.6, 12.8] | 12 [8, 16] |
| **Lipoprotein particle size** | | | | |
| VLDL particle size | 0.61 [0.28, 0.94] | -0.58 [-1.43, 0.27] | 0.56 [0.00, 1.12] | 1.7 [1.0, 2.5] |
| LDL particle size | -0.018  [-0.089, 0.054] | 0.21  [0.04, 0.38] | -0.11  [-0.22, 0.00] | -0.20  [-0.35, -0.05] |
| HDL particle size | 2.1 [1.8, 2.4] | 1.3 [0.6, 1.9] | 3.0 [2.6, 3.5] | 2.0 [1.4, 2.6] |
| **Apolipoproteins** | | | | |
| Apolipoprotein B | 29 [26, 32] | -12 [-19, -5] | 28 [24, 33] | 61 [55, 67] |
| Apolipoprotein A-I | 18 [16, 20] | -0.46 [-5.55, 4.64] | 24 [21, 27] | 24 [20, 29] |
| **Cholesterol** | | | | |
| Total C | 28 [26, 31] | -8.9 [-15.7, -2.1] | 31 [27, 36] | 51 [45, 57] |
| Non-HDL C | 31 [27, 34] | -15 [-24, -6] | 30 [25, 36] | 64 [56, 71] |
| Remnant C | 34 [31, 38] | -13 [-22, -5] | 33 [27, 38] | 70 [63, 77] |
| VLDL C | 45 [41, 50] | -13 [-23, -3] | 42 [35, 49] | 94 [85, 103] |
| IDL C | 24 [21, 27] | -13 [-21, -5] | 25 [19, 30] | 49 [42, 56] |
| LDL C | 28 [24, 31] | -17 [-26, -7] | 29 [22, 35] | 59 [51, 67] |
| HDL C | 22 [20, 25] | 3.3 [-3.8, 10.5] | 33 [28, 38] | 25 [19, 31] |
| HDL2 C | 25 [22, 29] | 5.2 [-4.3, 14.7] | 39 [33, 45] | 24 [16, 32] |
| HDL3 C | 15 [14, 16] | -0.72 [-3.46, 2.02] | 20 [18, 22] | 24 [22, 26] |
| Esterified C | 28 [25, 30] | -8.6 [-15.4, -1.7] | 31 [27, 36] | 49 [43, 55] |
| Free C | 28 [26, 31] | -9.6 [-16.4, -2.9] | 31 [27, 35] | 52 [46, 58] |
| Esterified C (%) | -0.049  [-0.250, 0.152] | 0.30  [-0.19, 0.79] | 0.046  [-0.277, 0.368] | -0.56  [-0.99, -0.14] |

| **Triglycerides** | | | | |
| --- | --- | --- | --- | --- |
| Total TG | 95 [89, 101] | -2.1 [-17.2, 13.0] | 94 [84, 103] | 180 [167, 193] |
| VLDL TG | 72 [64, 80] | -17 [-37, 3] | 62 [49, 74] | 158 [141, 175] |
| IDL TG | 116 [111, 121] | 11 [-1, 22] | 122 [115, 130] | 207 [197, 217] |
| LDL TG | 146 [140, 152] | 17 [3, 31] | 161 [152, 170] | 253 [241, 265] |
| HDL TG | 107 [102, 111] | 25 [14, 36] | 121 [114, 128] | 153 [144, 162] |
| **Phospholipids** | | | | |
| Total PL | 29 [27, 31] | -2.0 [-7.3, 3.4] | 33 [30, 37] | 45 [41, 50] |
| VLDL PL | 58 [53, 63] | -13 [-24, -1] | 53 [46, 61] | 117 [107, 127] |
| IDL PL | 32 [29, 35] | -9.6 [-17.1, -2.0] | 32 [27, 37] | 60 [53, 66] |
| LDL PL | 25 [23, 28] | -11 [-17, -4] | 27 [23, 31] | 50 [44, 55] |
| HDL PL | 22 [19, 24] | 5.4 [-1.2, 12.0] | 32 [27, 36] | 21 [16, 27] |
| Cholines | 36 [34, 38] | -0.54 [-6.57, 5.48] | 42 [39, 46] | 57 [52, 62] |
| Sphingomyelin | 27 [25, 29] | -1.1 [-6.5, 4.4] | 33 [29, 36] | 44 [39, 48] |
| Phosphoglycerides | 36 [34, 39] | -3.1 [-9.6, 3.5] | 43 [38, 47] | 58 [53, 64] |
| **Fatty acids** | | | | |
| Total FA | 40 [38, 43] | -2.7 [-9.1, 3.7] | 45 [41, 50] | 68 [62, 73] |
| Saturated FA | 45 [43, 48] | -1.9 [-8.7, 4.8] | 51 [46, 55] | 77 [71, 82] |
| MUFA | 53 [49, 56] | -2.3 [-10.6, 6.0] | 58 [53, 63] | 92 [85, 99] |
| PUFA | 28 [26, 30] | -3.6 [-9.6, 2.3] | 32 [28, 36] | 48 [43, 53] |
| Omega-6 FA | 27 [24, 29] | -5.2 [-11.1, 0.8] | 30 [26, 34] | 47 [42, 52] |
| Linoleic acid | 24 [21, 26] | -7.6 [-13.7, -1.4] | 26 [22, 30] | 44 [39, 49] |
| Omega-3 FA | 39 [35, 42] | 7.3 [-1.3, 15.8] | 49 [43, 55] | 57 [50, 64] |
| Docosahexaenoic acid | 56 [52, 60] | 13 [3, 23] | 70 [63, 76] | 85 [76, 93] |
| **Fatty acid ratios** | | | | |
| Saturated FA (%) | 3.2 [2.7, 3.8] | 0.72 [-0.62, 2.07] | 3.1 [2.2, 4.0] | 5.1 [3.9, 6.2] |
| MUFA (%) | 8.4 [7.2, 9.6] | 0.46 [-2.61, 3.52] | 8.6 [6.6, 10.6] | 15 [12, 17] |
| PUFA (%) | -8.4 [-9.3, -7.5] | -1.0 [-3.2, 1.2] | -8.5 [-10.0, -7.1] | -14 [-16, -13] |
| Omega-6 FA (%) | -9.6 [-10.5, -8.6] | -2.4 [-4.7, -0.2] | -10 [-12, -9] | -15 [-17, -13] |
| Linoleic acid (%) | -12 [-13, -11] | -4.6 [-7.4, -1.9] | -13 [-14, -11] | -17 [-19, -14] |
| Omega-3 FA (%) | 0.93 [-1.54, 3.41] | 11 [5, 17] | 3.3 [-0.6, 7.2] | -8.5 [-13.6, -3.4] |
| Docosahexaenoic acid (%) | 12 [10, 15] | 16 [9, 22] | 18 [13, 22] | 7.2 [1.4, 13.0] |
| Degree of unsaturation | -2.9 [-3.4, -2.4] | 0.84 [-0.38, 2.06] | -2.7 [-3.5, -1.9] | -6.5 [-7.5, -5.4] |
| **Amino acids** | | | | |
| Alanine | 7.5 [5.6, 9.4] | -6.7 [-12.0, -1.5] | 7.4 [4.0, 10.8] | 17 [13, 22] |
| Glutamine | -17 [-18, -15] | -19 [-24, -14] | -18 [-21, -15] | -17 [-21, -13] |
| Glycine | -17 [-20, -15] | -19 [-25, -12] | -21 [-25, -17] | -18 [-23, -12] |
| *Branched-chain amino acids* | | | | |
| Isoleucine | 1.8 [-1.4, 5.0] | -6.8 [-14.7, 1.1] | 0.85 [-4.23, 5.94] | 10 [3, 17] |
| Leucine | -1.4 [-3.8, 1.0] | -6.5 [-12.4, -0.6] | -3.0 [-6.8, 0.8] | 0.16 [-4.84, 5.17] |
| Valine | -13 [-15, -11] | -8.3 [-14.3, -2.3] | -16 [-19, -12] | -20 [-26, -15] |
| *Aromatic amino acids* | | | | |
| Phenylalanine | 23 [21, 25] | 7.6 [2.8, 12.4] | 24 [21, 27] | 32 [28, 36] |
| Tyrosine | -17 [-20, -15] | -15 [-22, -9] | -21 [-25, -16] | -19 [-25, -14] |
| Histidine | 13 [11, 15] | 1.7 [-3.5, 7.0] | 16 [13, 20] | 19 [15, 24] |
| **Glycolysis and gluconeogenesis** | | | | |
| Glucose | -7.8 [-9.4, -6.3] | -5.3 [-10.0, -0.5] | -7.9 [-10.8, -5.0] | -9.9 [-13.8, -6.0] |
| Lactate | 8.3 [5.1, 11.4] | -1.6 [-10.1, 6.8] | 10 [5, 15] | 13 [6, 20] |
| Pyruvate | 11 [7, 14] | -0.0052  [-8.8867, 8.8763] | 12  [6, 17] | 21  [13, 28] |
| Citrate | 0.45 [-1.62, 2.52] | -4.6 [-9.9, 0.7] | -0.84 [-4.32, 2.64] | 12 [8, 17] |
| Glycerol | -7.3 [-12.2, -2.4] | -14 [-26, -1] | -15 [-23, -7] | -16 [-27, -5] |
| **Ketone bodies** | | | | |
| Acetoacetate | -15 [-23, -8] | -18 [-39, 2] | -21 [-35, -8] | -18 [-35, 0] |
| Beta-hydroxybutyrate | 0.20 [-6.50, 6.91] | -9.1 [-26.0, 7.8] | -1.9 [-12.6, 8.8] | 9.8 [-4.3, 23.9] |
| **Miscellaneous** | | | | |
| Creatinine | -17 [-19, -15] | -18 [-23, -13] | -17 [-20, -13] | -13 [-18, -9] |
| Albumin | -9.2 [-10.4, -7.9] | -5.8 [-10.0, -1.5] | -10 [-13, -8] | -16 [-20, -12] |
| Acetate | -0.28 [-3.32, 2.76] | -11 [-18, -3] | 3.0 [-1.9, 7.9] | 4.1 [-2.3, 10.4] |
| **Inflammation markers** | | | | |
| Glycoprotein acetyls | 19 [17, 21] | 1.5 [-3.9, 6.9] | 20 [16, 23] | 29 [25, 34] |
| C-reactive protein | 169 [147, 192] | 76 [19, 134] | 199 [162, 236] | 165 [117, 214] |
| **Hormone related** | | | | |
| Insulin | 7.4 [1.9, 12.8] | -2.9 [-15.7, 10.0] | 3.1 [-5.4, 11.6] | 22 [12, 33] |
| Leptin | 50 [37, 63] | 34 [-13, 81] | 60 [36, 83] | 60 [28, 93] |
| Adiponectin | 1.1 [-7.3, 9.5] | 15 [-14, 44] | 5.0 [-9.3, 19.3] | -7.3 [-27.0, 12.4] |
| Vitamin D | -8.5 [-14.6, -2.5] | -2.3 [-17.9, 13.3] | -9.5 [-20.0, 1.1] | 11 [-3, 24] |
| SHBG | 483 [471, 496] | 218 [196, 239] | 527 [513, 541] | 697 [678, 715] |
| Testosterone | 93 [87, 98] | 115 [100, 130] | 100 [90, 110] | 130 [117, 143] |

The cross-sectional associations of pregnancy and pregnancy trimesters with 87 metabolic measures. The association magnitudes of pregnancy are reported as the metabolic difference between pregnant and non-pregnant women in relative to the metabolic concentrations of non-pregnant women. The association magnitudes of the trimesters are reported as the metabolic difference between the first, the second, and the third trimesters and non-pregnant women in relative to the metabolic concentration of non-pregnant women. The associations were adjusted for age. The study design and the abbreviations are as in Table S2.

**Table S5. Characteristics of the study participants during the pregnancy trimesters.**

| **Characteristics** | **NFBC 1966** | | | | **YFS** | | | |
| --- | --- | --- | --- | --- | --- | --- | --- | --- |
|  | Non-pregnant | First trimester | Second trimester | Third trimester | Non-pregnant | First trimester | Second trimester | Third trimester |
| Number | 1782 | 40 | 83 | 50 | 806 | 8 | 33 | 17 |
| Pregnant weeks | – | 9 [8-10] | 20 [17-24] | 32 [30-36] | – | 10 [7-11] | 19 [15-26] | 32 [31-35] |
| Age [year] | 31 (0) | 31 (0) | 31 (0) | 31 (0) | 32 (5) | 30 (3) | 31 (4) | 31 (5) |
| Weight [kg] | 65 (13) | 64 (9) | 67 (10) | 72 (13) | 67 (13) | 66 (10) | 71 (12) | 73 (13) |
| Waist [cm] | 79 (11) | 80 (11) | 86 (7) | 97 (7) | 80 (11) | 78 (6) | 84 (12) | 92 (5) |
| Hip [cm] | 97 (8) | 97 (7) | 96 (4) | 100 (6) | 100 (9) | 98 (7) | 103 (8) | 103 (5) |
| BMI [kg/m2] | 24 (4) | 23 (4) | 24 (3) | 26 (5) | 24 (5) | 24 (5) | 26 (4) | 27 (4) |
| Systolic blood pressure [mmHg] | 117 (10) | 118 (9) | 115 (9) | 113 (8) | 111 (10) | 109 (7) | 109 (12) | 109 (12) |
| Diastolic blood pressure [mmHg] | 73 (8) | 68 (10) | 64 (9) | 68 (10) | 68 (8) | 57 (9) | 60 (8) | 65 (9) |
| Smoking prevalence [%] | 38 | 24 | 26 | 20 | 20 | 12 | 9 | 6 |
| Alcohol usage [g/day] | 2.1  [0.6-5.7] | 0.9  [0.0-4.2] | 0.9  [0.0-2.6] | 1.0  [0.0-2.6] | 3.3  [0.0-8.2] | 0.0  [0.0-0.0] | 0.0  [0.0-0.0] | 0.0  [0.0-0.0] |
| Parity [n] | 2 [1-2] | 2 [1-2] | 1 [0-2] | 1 [1-2] | 1 [0-2] | 1 [1-2] | 1 [0-2] | 1 [0-1] |

Values are mean (SD) for normally distributed and median [interquartile range] for skewed variables. BMI: body mass index.

**Figure S1. Cross-section associations with and without the exclusion of breastfeeding women in YFS.** Metabolic concentrations of pregnant women (n=60) were compared to those of non-pregnant women (n=806) in YFS in 2001. The associations were adjusted for age. The metabolic associations with pregnancy were almost identical when 52 breastfeeding women were excluded from the non-pregnant women, suggesting minor confounding due to breastfeeding on the metabolic associations in this study setting.

**Figure S2A. Distributions of metabolic measures quantified via the NMR metabolomics platform in the three Finnish cohorts.**

**Figure S2B.**

**Figure S2C.**

**Figure S2D.**

**Figure S3A. Replication of cross-sectional and longitudinal associations between pregnancy and 87 metabolic measures.** The left panels shows the cross-sectional associations in the individual cohorts, including NFBC1966 (pregnant/non-pregnant=191/1782), YFS in 2001(pregnant/non-pregnant=60/806), and FINRISK1997 (pregnant/non-pregnant=71/1350). The associations were adjusted for age. The middle and right panels show the consistency of the longitudinal associations across 4-year (in YFS from 2007 to 2011), 6-year (from 2001 to 2007) and 10-year (from 2001 to 2011) follow-up. In the middle panel, women who were pregnant at the follow-up (but not at the baseline) were compared to those not being pregnant at both time points. In the right panel, women who were pregnant at baseline (but not at follow-up) were compared to those non-pregnant at both time points. There were 6, 18 and 5 women pregnant at follow-up in the 4-y, 6-y and 10-year follow-up; 23, 44 and 41 women were pregnant at baseline; and 623, 519 and 497 women were not pregnant at both time points, respectively.

**Figure S3B.**

**Figure S3C.**

**Figure 3D.**

**Figure S4. Cross-sectional associations between pregnancy and 87 metabolic measures with or without further adjustment of parity, BMI, smoking, alcohol usage and mean arterial pressure.** The associations were meta-analyzed across NFBC1966, YFS and FINRISK1997. Abbreviations are as in Table S1. Open and closed circles indicate P≥0.0008 and P<0.0008, respectively.

**Figure S5. Metabolic differences associated with pregnancy compared to those associated with higher non-VLDL-TG and higher total fatty acids (FA).** Non-VLDL-TG is defined as the sum of IDL-TG, LDL-TG and HDL-TG. The color scale indicates the metabolic differences (beta coefficients) associated with pregnancy and higher non-VLDL-TG and higher FA. The metabolic associations for pregnancy were defined as metabolic differences between pregnant and non-pregnant women. The metabolic associations were adjusted for age and meta-analysed for the three Finnish cohorts. To calculate the metabolic associations for higher non-VLDL-TG, the non-pregnant study participants were divided into two groups, one group with non-VLDL-TG concentration higher than the median and the other group with non-VLDL-TG concentration lower than the median. The metabolic associations were calculated as the metabolic differences across the metabolic profile between these two groups similar to comparing pregnant to non-pregnant women. The metabolic associations were adjusted for age. The associations were analysed in each individual cohort separately and then meta-analysed across all the 3 cohorts for 3938 non-pregnant women. The metabolic associations with higher FA were calculated in a similar fashion. The concentration differences between the higher and lower half were: 0.21 mmol/L (equivalent to 1.38 SDs) for non-VLDL-TG and 3.70 mmol/L (1.48 SDs) for FA.

| R^2^ (Goodness of fit) | Pregnancy vs non-VLDL-TG | Pregnancy vs FA | non-VLDL-TG vs FA |
| --- | --- | --- | --- |
| Entire metabolic profile | 0.41 | 0.38 | 0.87 |
| Lipids | 0.66 | 0.61 | 0.85 |
| Non-lipids | 0.00 | 0.01 | 0.92 |

The overall metabolic associations appear similar between the non-VLDL-TG and FA stratified groups of non-pregnant women (R2=0.87, shown in the table below). This is actually not unexpected since the circulating total fatty acids are strongly correlated with serum triglycerides (each triglyceride molecule carries 3 fatty acid chains). However, when comparing the metabolic signature of pregnancy to the metabolic association patterns of higher non-VLDL-TG or FA, similarities are only observed for the associations with apoB-lipoprotein –related measures (that include majority of circulating triglycerides and cholesterol as well as individual concentrations of multiple fatty acids). On the other hand, marked differences are noticed for HDL-related measures, fatty acid composition (that, in contradiction to fatty acid concentrations, are not necessarily dependent on circulating triglyceride concentration) and basically for all low-molecular-weight metabolites. Thereby these analyses clearly support an interpretation that many of the metabolic changes seen are likely specific for the pregnancy status. These interpretations are supported by the correlation analyses of the betas given below in the Table; R^2^ is used to summarise the similarity of the metabolic profiles.

**Figure S6. Longitudinal associations for women pregnant at follow-up with further adjustments.** The 6-year metabolic changes in women pregnant at follow-up (but not at baseline) were compared to those in women non-pregnant at both time points. The associations adjusted for baseline age were compared to those with further adjustments for baseline parity and 6-year change in BMI, smoking, alcohol usage and MAP. Abbreviations are as in Table S1. Open and closed circles indicate P≥0.0008 and P<0.0008, respectively.

**Figure S7. Longitudinal associations for women pregnant at baseline with further adjustments.** The 6-year metabolic changes in women pregnant at baseline (but not at follow-up) were compared to those of non-pregnant women at both time points. The associations adjusted for baseline age were compared to those with further adjustments for baseline parity and 6-year change in BMI, smoking, alcohol usage and MAP. Abbreviations are as in Table S1. Open and closed circles indicate P≥0.0008 and P<0.0008, respectively.

**Figure S8. Cross-sectional associations between the pregnancy trimesters and 87 metabolic measures in individual cohorts.** Pregnant women in their first, second and third trimester were compared to non-pregnant women. The associations were adjusted for age. Abbreviations are as in Table S1. Open and closed circles indicate P≥0.0008 and P<0.0008, respectively.

**Figure S9A. Cross-sectional associations of postpartum length with 85 metabolic measures.** Blood samples were collected at 31-y field study in 1997. Birth registry data from NFBC66 during the period from 1995 to 1997 were used. Women postpartum up to 2 months (n=28), 2 < months ≤ 4 (n=46), 4 < months ≤ 6 (n=42) and 6 < months ≤ 12 (n=123) were compared to those postpartum 1-3 years (n=389). The associations were adjusted for age.

**Figure S9B.** Please note that the correct effects size of pregnancy on SHBG is 2.6 SDs.

**Figure S10. Cross-sectional associations with and without the exclusion of women postpartum 0-6 months.** Metabolic concentrations of pregnant women (n=191) were compared to those of non-pregnant women (n=1782) in NFBC1966. The associations were adjusted for age. The metabolic associations with pregnancy were basically the same when 116 women, who had a delivery 0-6 months prior to blood sampling, were excluded from the non-pregnant women. This suggests the metabolic associations of pregnancy are very robust, and they are not affected by inclusion of women postpartum 0-6 months.

**Figure S11. Cross-sectional associations between pregnancy and 37 cytokines.** The cytokine concentrations in pregnant women (n=104) were compared to those of non-pregnant women (n=2217). The associations were adjusted for age. The measures available in both YFS in 2007 and FINRISK1997 are indicated with * and the associations meta-analysed for these two studies. Otherwise, the associations were calculated only from YFS in 2007. Abbreviations: IL, interleukin; IL-1ra: interleukin 1 receptor antagonist; IL-2rα: interleukin-2 receptor alpha; TNFα, tumor necrosis factor-alpha; TRAIL, TNF-related apoptosis inducing ligand; HGF, hepatocyte growth factor; βNGF, beta nerve growth factor; SCF, stem cell factor; SCGFβ, stem cell growth factor beta; GCSF, granulocyte colony-stimulating factor; VEGF, vascular endothelial growth factor; FGF basic, basic fibroblast growth factor; PDGF-BB, platelet derived growth factor BB; MCP-1, monocyte chemotactic protein-1; MIP–1α, macrophage inflammatory protein-1 alpha; MIP-1β, macrophage inflammatory protein-1 beta; CTACK, cutaneous T cell-attracting chemokine; GROα, growth regulated oncogene–alpha; MIG, monokine induced by interferon-gamma; IP–10, interferon gamma-induced protein 10; SDF1α, stromal cell-derived factor-1 alpha; MIF, macrophage migration inhibitory factor; IFNγ, interferon-gamma.
