# Supplementary material for: Metabolic profiling of pregnancy: cross-sectional and longitudinal evidence
Source: BMC Med. 2016 Dec 13;14:205. doi: 10.1186/s12916-016-0733-0 (PMC5153817; doi:10.1186/s12916-016-0733-0)
Supplement: Supplementary file 2 — Correlation network analysis. (PDF 7311 kb) [file 12916_2016_733_MOESM2_ESM.pdf]

Supplementary Material

Wang et al.

Metabolic Profiling of Pregnancy: Cross-sectional and Longitudinal Evidence

This file summarises the information related to the multivariate correlation network analysis conducted in this work. Description of the methodology and interpretation of the results are included together with a figure and two tables.

Supplementary methods and results

Network analysis.

Supplementary Figures

Figure S12. Visualisation of the various correlation networks; non-pregnant refers to non-pregnant women and pregnant for pregnant women. The difference refers to a network calculated based on the difference in the metabolic correlations between pregnant and non-pregnant women.

Supplementary Tables

Table S6. Node strengths for the various correlation networks; non-pregnant refers to non-pregnant women and pregnant for pregnant women. The difference refers to a network calculated based on the difference in the metabolic correlations between pregnant and non-pregnant women.

Table S7. Mean correlation magnitudes within and between node communities in the various correlation networks; non-pregnant refers to non-pregnant women and pregnant for pregnant women. The difference refers to a network calculated based on the difference in the metabolic correlations between pregnant and non-pregnant women.

Network analysis

Each of the 87 metabolic measures was adjusted for age in the individual cohort. Pair-wise Spearman correlation coefficients between the residuals of the 87 metabolic variables were estimated for non-pregnant and pregnant women in each three cohorts. The overall coefficients for non-pregnant and pregnant women were calculated as the means of the cohort-specific coefficients, weighted by the number of participants. For the network analyses, squared coefficients were used, as they indicate the proportion variance explained and are less sensitive to spurious values close to zero, and they are also guaranteed to be non-negative. Differences in correlation were estimated by restoring the sign for the direction of the correlation to the squared coefficients, and then subtracting the values for the non-pregnant from the values for the pregnant women. Again, this approach was chosen to reduce the effects of spurious signals near zero from sampling noise. In the networks, the nodes represent the metabolic variables and the edge weights indicate the pair-wise correlations (or the differences in correlation associated with pregnancy).

Network visualisations were created in multiple steps. First, agglomerative community detection was performed on the non-pregnant correlation network to group strongly inter-correlated variables. This step was necessary to make the figures easier to interpret via the community structure that reflects the inherent correlation patterns in the population. Next, the node positions for the graphical presentation were optimised by a force-directed algorithm according to the difference network and the community structure for the non-pregnant women. This approach was chosen to ensure clearer presentation of the differences in correlations while maintaining the correlation patterns of the population. For the final three plots (Figure S12A-C, Additional file 2), edge widths and colours were assigned according to the direction and magnitude of the correlation coefficients (network for the non-pregnant and pregnant women in plots B and C, respectively) or by the differences in correlation (plot A).

The strength of a node was defined as the sum of the adjacent edge weights (i.e. squared correlation coefficients). For the pregnancy comparison, the node strengths in the network for non-pregnant women were subtracted from the node strengths in the network for pregnant women to indicate those variables that were the most associated with pregnancy. However, unadjusted differences in node strengths were confounded by situations where the strength was very low in the network for non-pregnant women (e.g. testosterone), and modestly higher in the network for pregnant women – mostly due to the higher sampling noise from the smaller participant count of pregnant women. For this reason, adjusted proportional changes in node strengths in the difference network were estimated by the formula $\boldsymbol{d} = |\boldsymbol{s_1} - \boldsymbol{s_0}|/(\boldsymbol{s_0} + \mu)$, where $\boldsymbol{s_0}$ and $\boldsymbol{s_1}$ are vectors that contain the node strengths from networks for the non-pregnant and pregnant women, and $\mu = \text{median}(|\boldsymbol{s_1} - \boldsymbol{s_0}|)$ is the adjustment factor. The pregnancy-associated differences in the node strength with and without adjustment are listed in Table S6 (Additional file 2). In the network plots, the radius of the node symbol indicates the strength (networks for the non-pregnant and pregnant women) or the adjusted pregnancy-associated difference in topology (difference network).

In general, a higher degree of pair-wise correlation was observed in the pregnant (mean Spearman $|r| = 0.39$) compared to the non-pregnant women (mean $|r| = 0.29$). The node communities in the network were assigned based on the topology, and this was reflected by the community-level mean correlations: the mean correlation

magnitude was larger within the communities compared to between communities (diagonal vs. non-diagonal elements in the first two sections in Table S7, Additional file 2). The mean differences behaved in an opposite way: the diagonal values in the third section in Table S7 (Additional file 2) were smaller compared to the non-diagonal values. These observations confirm the usefulness of the community detection as a means to simplify the presentation; the communities contain mostly variables that are bound by basic biochemistry (as opposed to biological regulation), thus reducing the distraction from non-biological correlations. Although biological phenomena cannot be fully disentangled from the statistical biases associated with sample count and selection, the topological shift is also compatible with pregnancy acting as a metabolic stressor that amplifies any intrinsic patterns of metabolism in women. Of note, 28% of all correlation coefficients were significantly different ($P < 5.7 \times 10^{-4}$ for a single test, $P < 0.05$ for 87 independent tests) between the networks for pregnant and non-pregnant women.

Sex-hormone binding globulin (SHBG) showed a substantial difference in node strength between the pregnant and non-pregnant women ($s_1 = 14.3$ vs. $s_0 = 2.52$, $(s_1 - s_0)/s_0 = +469\%$, $d = 94\%$). SHBG was also the variable that showed the greatest pregnancy-associated increase of +483% in absolute concentration. From a mathematical perspective, this effect of the expanded concentration range (i.e. increased “amplitude”) translates into lower signal-to-noise ratio that may partly explain the correlation differences, however, the stronger regulation of metabolism by hormonal changes is also a plausible biological explanation for the finding. Pronounced node differences were also noted for triglycerides in several lipoprotein categories (e.g. HDL, LDL and IDL) in the “Triglyceride enrichment” community. These nodes, together with alanine and phenylalanine in the “Amino acid” community, and measures in the “Fatty acid composition” community all showed a similar pattern of higher inter-community connectivity in the network for pregnant women compared to the network for non-pregnant women. The correlation between leucine and valine was one of the few intra-community edges that exhibited a noticeable pregnancy association ($r = 0.40$ in pregnant vs. $r = 0.75$ in non-pregnant women).

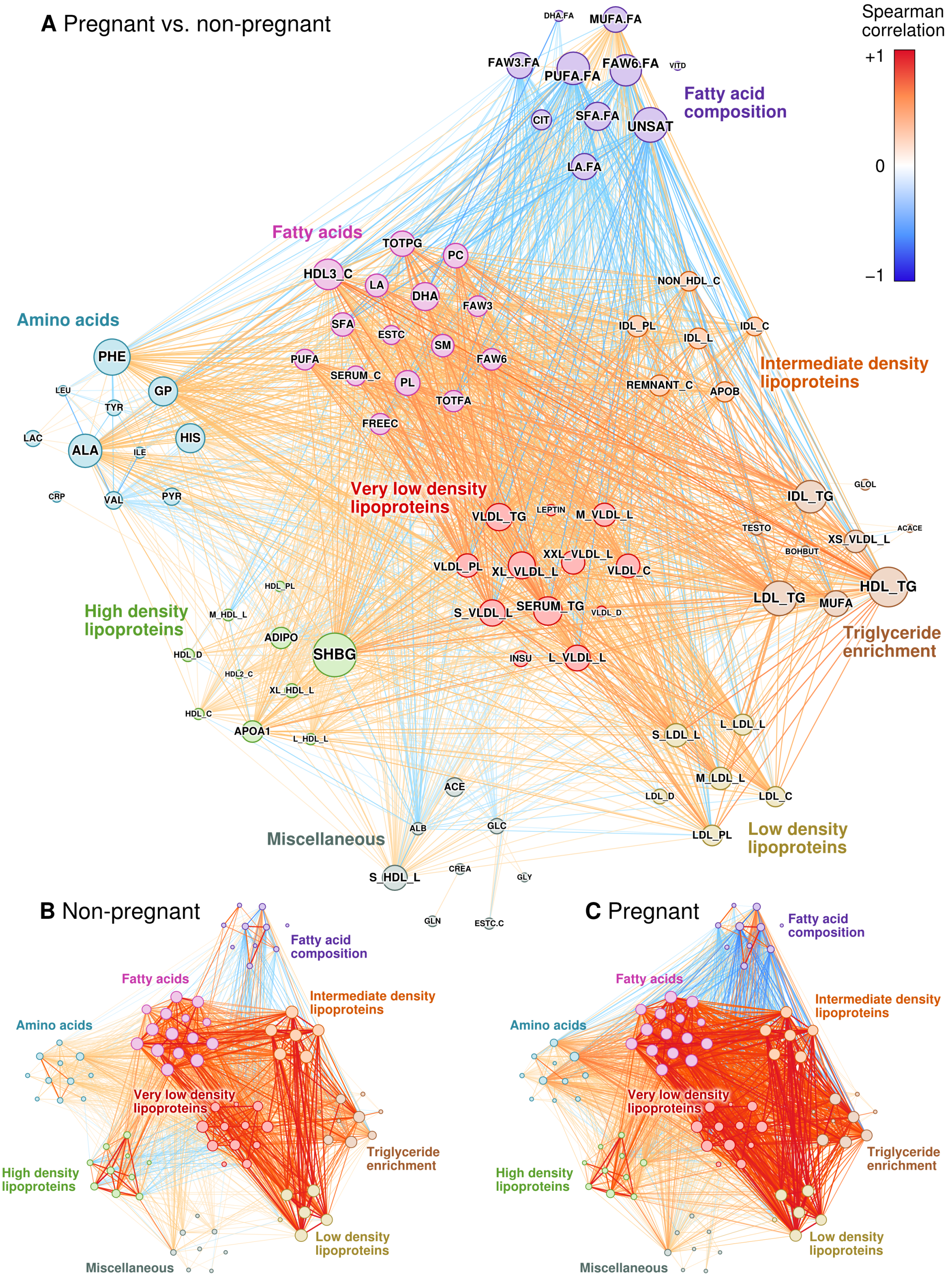

Figure S12. Visualisation of the multivariable correlation networks.

The nodes represent the metabolic variables and the edge weights indicate the pair-wise metabolic correlations. Non-pregnant refers to non-pregnant women (plot B) and pregnant for pregnant women (plot C). Plot A illustrates the network calculated based on the difference in the metabolic correlations between pregnant and non-pregnant women.

Table S6. Node strengths in the various correlation networks.

NODE	S_0 (node strength in the network for non-pregnant women)	S_1 (node strength in the network for pregnant women)	DIFF (difference in node strength, without adjusting for sampling noise)	DIFF_adjusted (difference in node strength, adjusted for sampling noise)
SHBG	2.52	14.33	469%	94%
HDL_TG	13.96	34.01	144%	84%
PHE	6.34	18.54	192%	74%
UNSAT	5.79	16.97	193%	71%
LDL_TG	16.87	35.84	112%	70%
ALA	7.38	19.02	158%	67%
IDL_TG	17.42	35.25	102%	65%
PUFA.FA	8.81	21.03	139%	65%
FAW6.FA	7.97	19.11	140%	62%
HDL3_C	15.76	31.21	98%	60%
HIS	3.27	10.70	227%	56%
GP	12.09	24.39	102%	56%
SERUM_TG	19.31	35.23	82%	54%
SFA.FA	1.65	7.89	377%	53%
DHA	12.74	24.82	95%	53%
VLDL_TG	16.54	30.09	82%	51%
XL_VLDL_L	13.68	25.62	87%	50%
S_VLDL_L	19.80	34.27	73%	49%
LA.FA	6.58	14.58	122%	48%
FAW3.FA	3.54	9.98	182%	47%
L_VLDL_L	15.36	27.34	78%	47%
MUFA	22.22	37.41	68%	47%
MUFA.FA	9.00	17.86	99%	47%
TOTPG	21.76	36.38	67%	46%
PL	20.78	34.65	67%	45%
S_HDL_L	6.83	14.41	111%	45%
PC	21.68	35.63	64%	44%
VLDL_C	21.39	34.39	61%	41%
SFA	23.68	37.49	58%	41%
VLDL_PL	22.52	35.81	59%	41%
XXL_VLDL_L	12.96	22.31	72%	41%
S_LDL_L	22.25	35.34	59%	41%
M_VLDL_L	17.48	28.53	63%	40%
LA	20.99	33.18	58%	39%
XS_VLDL_L	21.95	34.34	56%	39%
SM	21.24	33.26	57%	38%
M_LDL_L	22.69	35.26	55%	38%
FAW6	23.07	34.98	52%	36%
APOA1	13.38	21.76	63%	36%
IDL_PL	23.11	34.89	51%	36%
ADIPO	2.38	6.79	185%	36%
TOTFA	25.72	38.39	49%	35%
FREEC	23.92	35.76	49%	35%
REMNANT_C	23.87	35.63	49%	35%
L_LDL_L	23.56	35.20	49%	35%
IDL_L	23.48	34.93	49%	34%

PUFA	23.54	34.95	48%	34%
LDL_PL	24.42	36.06	48%	34%
FAW3	12.71	20.34	60%	34%
LDL_C	21.92	32.32	47%	33%
APOB	25.16	36.58	45%	32%
CIT	1.45	5.12	254%	32%
SERUM_C	24.79	35.65	44%	31%
NON_HDL_C	24.38	34.95	43%	31%
ESTC	24.48	34.51	41%	29%
IDL_C	22.18	31.38	42%	29%
ACE	1.30	4.51	247%	28%
VAL	6.85	2.54	-63%	26%
PYR	3.48	6.82	96%	25%
LAC	2.99	5.79	94%	22%
TYR	4.44	1.44	-68%	21%
INSU	3.97	6.83	72%	20%
GLC	4.61	1.73	-62%	20%
LDL_D	2.83	5.17	82%	18%
XL_HDL_L	6.58	9.27	41%	16%
TESTO	0.25	1.72	593%	14%
HDL_D	7.36	4.99	-32%	14%
ALB	5.93	4.02	-32%	12%
M_HDL_L	7.48	5.69	-24%	10%
LEPTIN	3.93	5.19	32%	9%
L_HDL_L	9.85	11.63	18%	9%
HDL_C	11.23	13.06	16%	9%
ESTC.C	1.29	2.26	75%	9%
ILE	9.10	10.74	18%	9%
DHA.FA	3.28	4.34	32%	8%
GLOL	2.64	3.61	37%	8%
CREA	2.33	1.40	-40%	8%
GLN	2.19	1.29	-41%	7%
BOHBUT	2.18	3.07	41%	7%
CRP	2.50	3.38	35%	7%
VLDL_D	11.16	12.66	13%	7%
HDL_PL	9.83	11.15	13%	7%
LEU	9.68	8.45	-13%	6%
GLY	1.06	1.36	29%	3%
VITD	0.29	0.57	92%	3%
HDL2_C	10.85	10.44	-4%	2%
ACACE	2.13	2.20	3%	1%

Table S7: Mean correlation magnitudes within and between node communities in the various correlation networks.

Mean correlation magnitudes within and between node communities in non-pregnant women									
	Misc	TG	HDL	IDL	AA	FA%	LDL	VLDL	FA
Misc: Miscellaneous	0.31	0.13	0.16	0.17	0.18	0.07	0.18	0.07	0.23
TG: Triglyceride enrichment	0.13	0.38	0.10	0.40	0.21	0.18	0.34	0.37	0.35
HDL: High density lipoproteins	0.16	0.10	0.60	0.11	0.13	0.15	0.11	0.29	0.31
IDL: Intermediate density lipoproteins	0.17	0.40	0.11	0.96	0.27	0.08	0.79	0.46	0.71
AA: Amino acids	0.18	0.21	0.13	0.27	0.43	0.12	0.21	0.27	0.27
FA%: Fatty acid composition	0.07	0.18	0.15	0.08	0.12	0.41	0.05	0.27	0.12
LDL: Low density lipoproteins	0.18	0.34	0.11	0.79	0.21	0.05	0.82	0.31	0.67
VLDL: Very low density lipoproteins	0.07	0.37	0.29	0.46	0.27	0.27	0.31	0.71	0.29
FA: Fatty acids	0.23	0.35	0.31	0.71	0.27	0.12	0.67	0.29	0.80

Mean correlation magnitudes within and between node communities in pregnant women									
	Misc	TG	HDL	IDL	AA	FA%	LDL	VLDL	FA
Misc: Miscellaneous	0.29	0.16	0.15	0.17	0.18	0.14	0.15	0.15	0.16
TG: Triglyceride enrichment	0.16	0.48	0.22	0.55	0.29	0.31	0.48	0.46	0.53
HDL: High density lipoproteins	0.15	0.22	0.57	0.29	0.19	0.15	0.30	0.18	0.45
IDL: Intermediate density lipoproteins	0.17	0.55	0.29	0.97	0.34	0.34	0.86	0.65	0.86
AA: Amino acids	0.18	0.29	0.19	0.34	0.40	0.23	0.30	0.35	0.35
FA%: Fatty acid composition	0.14	0.31	0.15	0.34	0.23	0.49	0.29	0.44	0.33
LDL: Low density lipoproteins	0.15	0.48	0.30	0.86	0.30	0.29	0.83	0.56	0.78
VLDL: Very low density lipoproteins	0.15	0.46	0.18	0.65	0.35	0.44	0.56	0.73	0.57
FA: Fatty acids	0.16	0.53	0.45	0.86	0.35	0.33	0.78	0.57	0.89

Mean differences in community-level correlation between pregnant and non-pregnant women									
	Misc	TG	HDL	IDL	AA	FA%	LDL	VLDL	FA
Misc: Miscellaneous	0.09	0.14	0.12	0.19	0.10	0.10	0.16	0.14	0.20
TG: Triglyceride enrichment	0.14	0.14	0.22	0.17	0.16	0.21	0.16	0.19	0.19
HDL: High density lipoproteins	0.12	0.22	0.07	0.27	0.17	0.20	0.22	0.35	0.17
IDL: Intermediate density lipoproteins	0.19	0.17	0.27	0.02	0.17	0.32	0.06	0.19	0.14
AA: Amino acids	0.10	0.16	0.17	0.17	0.10	0.17	0.17	0.17	0.19
FAcomp: Fatty acid composition	0.10	0.21	0.20	0.32	0.17	0.13	0.31	0.22	0.34
LDL: Low density lipoproteins	0.16	0.16	0.22	0.06	0.17	0.31	0.02	0.26	0.12
VLDL: Very low density lipoproteins	0.14	0.19	0.35	0.19	0.17	0.22	0.26	0.05	0.28
FA: Fatty acids	0.20	0.19	0.17	0.14	0.19	0.34	0.12	0.28	0.10
