# Supplementary material for: Metabolic profiling of pregnancy: cross-sectional and longitudinal evidence
Source: BMC Med. 2016 Dec 13;14:205. doi: 10.1186/s12916-016-0733-0 (PMC5153817; doi:10.1186/s12916-016-0733-0)
Supplement: Supplementary file 3 — Authors’ response to reviewers. (PDF 7434 kb) [file 12916_2016_733_MOESM3_ESM.pdf]

BMED-D-16-00718
Metabolic Profiling of Pregnancy: Cross-sectional and Longitudinal Evidence

Below we provide detailed responses to the points raised by the reviewers and the editor. Responses are written in blue and the changes made to the manuscript are shown in red. The original comments are given in black. As part of the revision package, a comparison Word document of the revised manuscript is included in which track-changes markings with respect to the original manuscript are given.

Comments from reviewers:
Reviewer #3 (Antonio Barros):
The manuscript by Wang et al. seeks to establish a metabolic pattern in pregnancy. For that purpose the authors rely on cross-sectional and longitudinal studied to highlight the relevant alterations.
The high complexity of the subject demands a cautionary approach for causal effects. The manuscript is clear and well structured. However, several issues should be addressed before any consideration on publication.

We do agree with the reviewer that it (always) requires caution in making causal conclusions via observational studies. Randomized controlled trial (RCT) design being the well-known gold standard in inferring causality in the area of health sciences. However, to address important research questions related to the impact of pregnancy in humans on physiology can only be done observationally. Mendelian randomization (the use of genetic variants as instrumental variables to improve causal inference) is also not possible in this context as genetic variants that influence becoming pregnant at any given time are not known. Given these constraints the best causal approach to address our objective is a prospective study in which a group of women change pregnancy status over time, which is what we present here (alongside a larger cross-sectional study and comparison between the two designs).

Given this, we feel that it is appropriate to suggest that pregnancy causes the observed metabolic aberrations.

1) Lines 232-233. Why the usage of log transformation of data; the authors should show the distribution of a relevant marker - for illustrational purposes.

An assumption of linear regression is that the residuals from the model are normally distributed. The primary determination of this is the distribution of the outcome (in our study the metabolites). Decisions regarding whether or not to transform outcome variables are a balance between avoiding important violation of model assumptions and being able to provide results that are readily interpretable. As many metabolites are markedly non-normal it has been recommended by some that these should be long transformed (Meikle et al. Int J Epidemiol. 2016 Jun 10. pii:dyw112). In the Figure below, for circulating triglyceride concentrations, it is evident

from the left panel of the Figure that the distributions are positively skewed (for both pregnant and non-pregnant women). The group with the higher mean (pregnant women) has a proportionally larger standard deviation as well. Both characteristics suggest that a logarithmic transformation could be used to make the data more symmetric and homoscedastic. The right panel of the Figure below demonstrates that log-transformation does achieve this purpose and works towards making the confidence intervals more reliable.

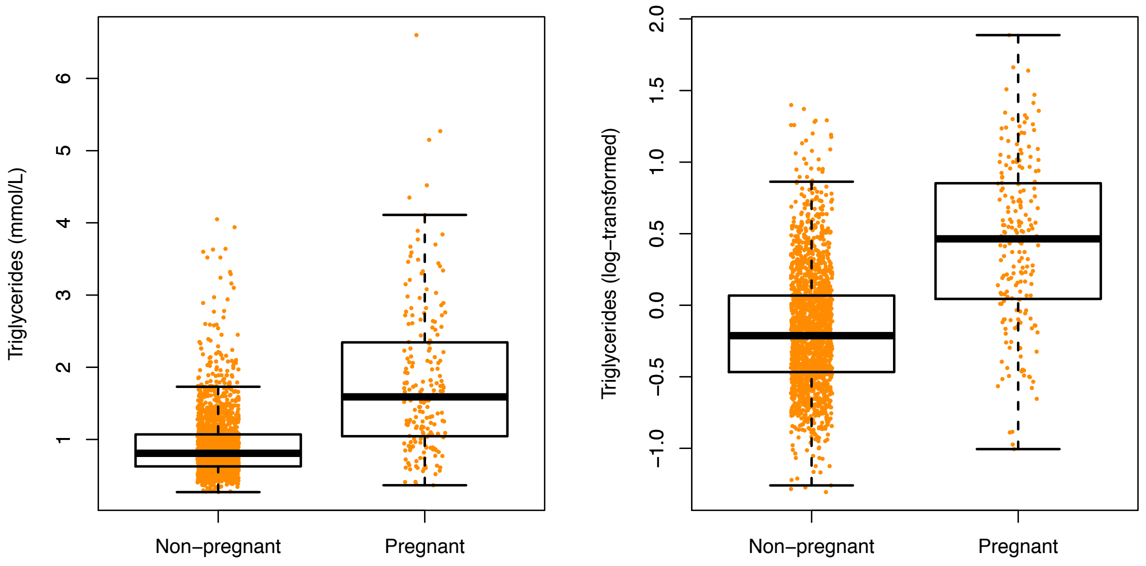

We have now added the distributions of the metabolic measures quantified by the NMR metabolomics platform in the supplement (Figure S1, Additional file 1). We have also justified the application of log-transformation in the statistical analysis section (page 9).

2) The authors have performed a PCA to establish a criterion for p-value correction. It is not clear on which data that was performed (number of samples; number of variables - I suppose 124) etc.

We thank the reviewer for highlighting this issue. The PC analyses were performed on each individual cohort. In each cohort, all women who had complete data on the metabolic measures were used in the analysis. The number of samples and number of available variables are listed in the Table below. Since the number of variables available varies across the cohorts, the number of PCAs needed to explain at least 99% of the variation in the data also varies. The YFS contains almost the complete set of variables, and was thus used to calculate the maximum number of independent variables (i.e., 61) for the statistical testing. Therefore, P values less than (0.05/61) were considered statistically significant after multiple testing correction. With the current threshold (0.05/61), 75 out of 87 metabolic measures and 9 out of 37 cytokines were statistically significant in the meta-analysis of cross-sectional associations. In fact, here, with a more stringent Bonferroni correction (0.05/124), the numbers of statistically significant metabolic measures remain the same.

We have now added details on the determination of the number of independent variables for the statistical testing and denoted the number of PCs for each cohort in the supplement (Table S1, Additional file 1). The exact P values for each metabolic association are now also reported in Table S2 (Additional file 1).

**Cohort**	**Sample size**	**Maximum number of variables available**	**Number of PCs explains at least 99% of variation in the data**
NFBC 1966	1349	85<br><br>(metabolic measures)	34
YFS<br>(2007 survey)	1133	121<br><br>(84 metabolic measures + 37cytokines)	61
FINRISK1997	2872	105<br><br>(86 metabolic measures + 19cytokines)	50

3) The authors used a linear regression to establish a relationship between biomarker measure and pregnancy outcome. Since the pregnancy status is a categorical variable why not the use of a logit regression instead?

The main aim of this work is to study the effects of pregnancy on systemic metabolism. That is, how pregnancy would potentially affect the concentrations of circulating metabolites and lipids. Therefore, pregnancy is the exposure and metabolites are the outcome measures, following a sound biological assumption that pregnancy precedes (causes) the metabolic changes. We do not think that a reversed assumption would be appropriate (or that a metabolic diagnostics for pregnancy would be relevant). Thus, our outcome measures are continuous variables and thus linear regression analysis is the appropriate choice.

4) Were all woman healthy?

We excluded from the analyses those women (whether pregnant or not) who had high fasting glucose or high blood pressure, at levels indicative of diabetes or hypertension, respectively. Although we cannot be certain that we have excluded all cases of gestational diabetes or hypertensive disorders of pregnancy, the analysis of young (mean age 31y) general population-based cohorts suggests that our participants are apparently healthy and our results are likely to be generalisable to most (European origin) women of reproductive age. This point is acknowledged in the limitations of the present study in the discussion section of the manuscript.

5) For such a complex study it would be interesting and helpful to perform a network analysis of the pertinent metabolites; in addition - and due to the multivariate core nature of the data a multivariate approach should be taken to fully address the intricate metabolomic relationships.

As emphasized above, the main aim of this work is to study the effects of pregnancy on systemic metabolism. A standard epidemiological approach was used, i.e., we analysed and aimed to understand how circulating metabolite, lipid and lipoprotein concentrations differ between women who are pregnant and those who are not (and longitudinally how they differ in the same women as they change pregnancy status). As circulating maternal metabolites are the key nutrients for the fetus development and growth, it is important to increase our understanding on how and to what extent maternal metabolism changes during the course of pregnancy, throughout the trimesters and postpartum. The linear regression analyses conducted in this work are a standard epidemiological way to address these questions. Importantly, the analyses conducted facilitate a clear and straightforward biological interpretation.

However, we do understand the interest of the reviewer for assessing potential changes in metabolic associations due to pregnancy. One potential tool for doing this is a metabolic (correlation) network as suggested by the reviewer and we anticipate that there are general interests in (multivariate correlation) network analyses, particularly in research fields focusing more on the statistical and analytical aspects of metabolomics. Whilst we feel keeping our main results as they are currently presented, we can see that analyses taking account of correlations between the metabolites would have some value. We have, therefore, performed multivariate correlation network analyses to assess potential differences in metabolic associations between pregnant and non-pregnant women. We have now added a new supplement material (Additional file 2) that contains methodological details of how we have done this, a Figure showing the results and a discussion of the interpretation of the key results. We refer to these additional analyses in the main paper (page 13). Briefly, in the Figure (which we also show as part of this response on the next page), plot A shows the difference in the metabolic correlation networks between pregnant and non-pregnant women and plots B and C illustrate the original correlation network for the non-pregnant and pregnant women, respectively. In general, a higher degree of pair-wise correlations was observed in pregnant (mean Spearman $|r| = 0.39$) compared to non-pregnant women (mean $|r| = 0.29$). A pronounced node difference was noted in SHBG, together with triglycerides in multiple lipoprotein categories ("Triglyceride enrichment" community), alanine and phenylalanine ("Amino acid"), and various fatty acids ("Fatty acid composition"). These nodes shared a similar pattern of higher inter-community connectivity for the pregnant than for the non-pregnant women. In general, the correlation network analyses suggest that pregnancy is acting as a metabolic stressor that amplifies any intrinsic patterns of metabolism in women. The metabolic results are elaborated more in the added supplement material (Additional file 2).

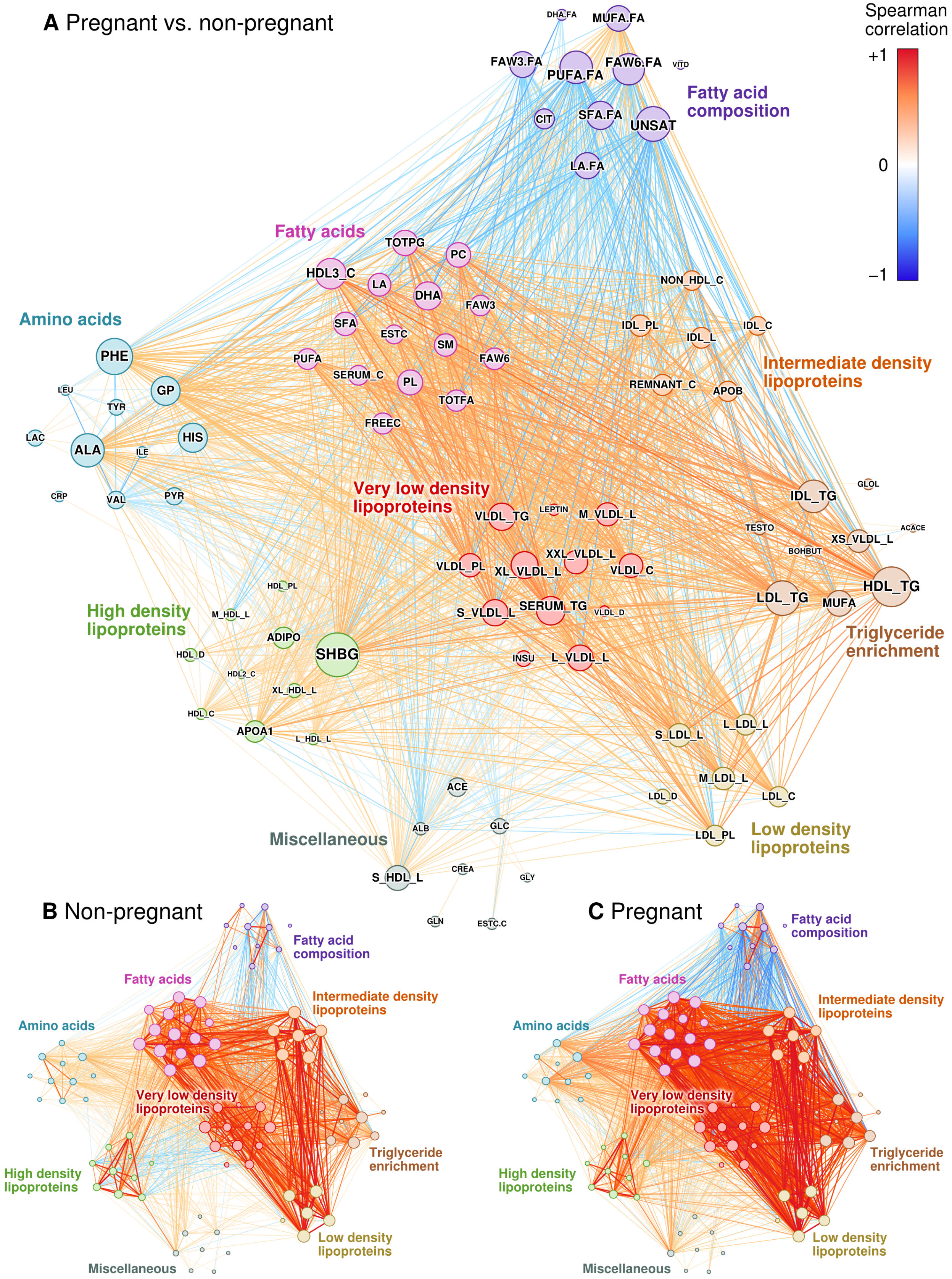
A Pregnant vs. non-pregnant
Spearman correlation
+1
0
−1
Fatty acid composition
Fatty acids
Amino acids
Intermediate density lipoproteins
Very low density lipoproteins
High density lipoproteins
Triglyceride enrichment
Miscellaneous
Low density lipoproteins
MUFA.FA
FAW3.FA
PUFA.FA
FAW6.FA
CIT
SFA.FA
UNSAT
LA.FA
TOTPG
PC
HDL3_C
LA
DHA
FAW3
SFA
ESTC
SM
PUFA
FAW6
SERUM_C
PL
TOTFA
FREEC
NON_HDL_C
IDL_PL
IDL_L
IDL_C
REMNANT_C
APOB
PHE
GP
TYR
ALA
HIS
VAL
PYR
IDL_TG
VLDL_TG
M_VLDL_L
XS_VLDL_L
XXL_VLDL_L
VLDL_PL
XL_VLDL_L
VLDL_C
HDL_TG
LDL_TG
MUFA
S_VLDL_L
SERUM_TG
SHBG
ADIPO
L_VLDL_L
APOA1
S_LDL_L
L_LDL_L
M_LDL_L
LDL_C
ACE
LDL_PL
S_HDL_L
B Non-pregnant
C Pregnant

6) The last sentence of the conclusion should be supported by the results; this is not the case for the current form of the manuscript.
In conclusion I do think that the wealthy data available for this work should be further explored as many interesting relationships should be possible to infer.

We are pleased for the positive comments of the reviewer for the wealth of the metabolic data. According to the reviewer's suggestion, we have now further explored the data with multivariate correlation network analyses and added a new supplement referring to these (Additional file 2).

We also modified the last concluding sentence to be more reflective of the actual results. The modified version now reads 'These findings provide a comprehensive foundation to the systemic molecular understanding of maternal metabolism during pregnancy.'

Reviewer #4 (Hector Keun):
This report describes analyses of the differences in NMR profiles and circulating inflammatory marker in blood serum between pregnant and non-pregnant women. In defining the signature differences related to pregnancy. Multiple cohorts are used and cross-sectional and longitudinal differences are compared. The signature effects described are convincing, particularly because of the high level of agreement between cross-sectional and longitudinal differences.

We are most pleased for these positive comments that capture the essence of the study design and results.

Minor essential revisions:
1. No accession number for the raw or processed data is given - please indicate in which repository these data will be accessible? If not could the authors give any timeline for release?

Metabolic and clinical data are available according to the rules of each individual cohort and can be requested from the Institutional Data Access Committees of the Northern Finland Birth Cohort Studies (University of Oulu, Finland), the Cardiovascular Risk in Young Finns Study (University of Turku, Finland), and the FINRISK study committee at the National Institute for Health and Welfare (Helsinki, Finland). This is now clarified in the section of 'availability of data and materials' under the heading of 'Declaration'.

2. While I fully appreciate the rationale for setting the number of tests for multiple testing correction to 61 via PCA analysis, I think some purists may not and hence if these data are not to be made public then a supplementary table indicating which metabolic differences associated with pregnancy were significant after Bonferroni would be useful for some, together with the effect sizes and confidence intervals given in full.

We acknowledge the reviewer for this comment. In general, the Bonferroni correction gives too conservative results in the case of correlated variables, as it treats each variable as an independent test; thus, using the number of independent variables (calculated by PCA) instead of the sum of all the original (correlated) variables is a straightforward solution to minimize both false positives and false negatives (Kujala et al. Circulation. 2013;127:340-8). In this particular case, however, it happened that the number of statistically significant associations is exactly the same at the PCA-modified ($P<0.05/61$) and conservative Bonferroni ($P<0.05/124$) level of significance.

To clarify the details, we have now added details on the determination of the number of independent variables for the statistical testing and denoted the number of PCs for each cohort in the supplement (Table S1, Additional file 1). The metabolic differences in SD units, together with the confidence interval and exact P values are now reported in Table S2 (Additional file 1). In the supplement, we also report the metabolic differences associated with pregnancy in both absolute physiological units (now Table S3, Additional file 1) and in percentage differences (now Table S4, Additional file 1); both effect sizes and confidence intervals are provided.

3. Because the pregnancy signature is so dominated by A) large increases in IDL, LDL & HDL triglycerides and B) total fatty acids a key question is how specific the other parts of the signature are to pregnancy. If non-pregnant women are stratified by A or B do we see changes in other parameters e.g. amino acid changes or the degree of unsaturation that mirror the pregnancy signature? If so, then the specificity of some of these effects is called into question, if not, there is further evidence in favour of the manuscript's findings from a different perspective. These analyses should be included and discussed in full within the main body of the manuscript.

The reviewer raises an interesting proposition, namely that other metabolic effects may not be independent of the four with the largest effects (IDL, LDL & HDL triglycerides and total fatty acids). To assess this we compared the metabolic associations of pregnancy to those associated with higher IDL-TG + LDL-TG + HDL-TG (non-VLDL-TG) and total fatty acids (FA) in non-pregnant women. To calculate the metabolic associations of higher non-VLDL-TG, the non-pregnant study participants were divided into two groups, one group with non-VLDL-TG concentration higher than the median and the other group with non-VLDL-TG concentration lower than the median. The metabolic associations were calculated as the metabolic differences across the metabolic profile between these two groups similar to comparing pregnant to non-pregnant women. The metabolic associations were adjusted for age. The associations were analysed in each individual cohort separately and then meta-analysed across all the 3 cohorts for 3938 non-pregnant women. The metabolic associations with higher FA were calculated in a similar fashion. The concentration differences between the higher and lower half were: 0.21 mmol/L (equivalent to 1.38 SDs) for non-VLDL-TG and 3.70 mmol/L (1.48 SDs) for FA.

The metabolic differences (the beta coefficients) from the analyses described above are shown in the heatmap figure below. The overall metabolic associations appear similar between the non-VLDL-TG and FA stratified groups of non-pregnant women ($R^2=0.87$, shown in the table below).

This is actually not unexpected since the circulating total fatty acids are strongly correlated with serum triglycerides (each triglyceride molecule carries 3 fatty acid chains). However, when comparing the metabolic signature of pregnancy to the metabolic association patterns of higher non-VLDL-TG or FA, similarities are only observed for the associations with apoB-lipoprotein – related measures (that include majority of circulating triglycerides and cholesterol as well as individual concentrations of multiple fatty acids). On the other hand, marked differences are noticed for HDL-related measures, fatty acid composition (that, in contradiction to fatty acid concentrations, are not necessarily dependent on circulating triglyceride concentration) and basically for all low-molecular-weight metabolites. Thereby these analyses, suggested by the reviewer, clearly support an interpretation that many of the metabolic changes seen are likely specific for the pregnancy status. We have now added the heatmap and the table shown below in the supplement (Figure S5, Additional file 1) and discussed this issue in the main text of the manuscript (page 13). In the table R^2 is used to summarise the similarity of the metabolic profiles.

R^2 (Goodness of fit)	Pregnancy vs non-VLDL-TG	Pregnancy vs FA	non-VLDL-TG vs FA
Entire metabolic profile	0.41	0.38	0.87
Lipids	0.66	0.61	0.85
Non-lipids	0.00	0.01	0.92

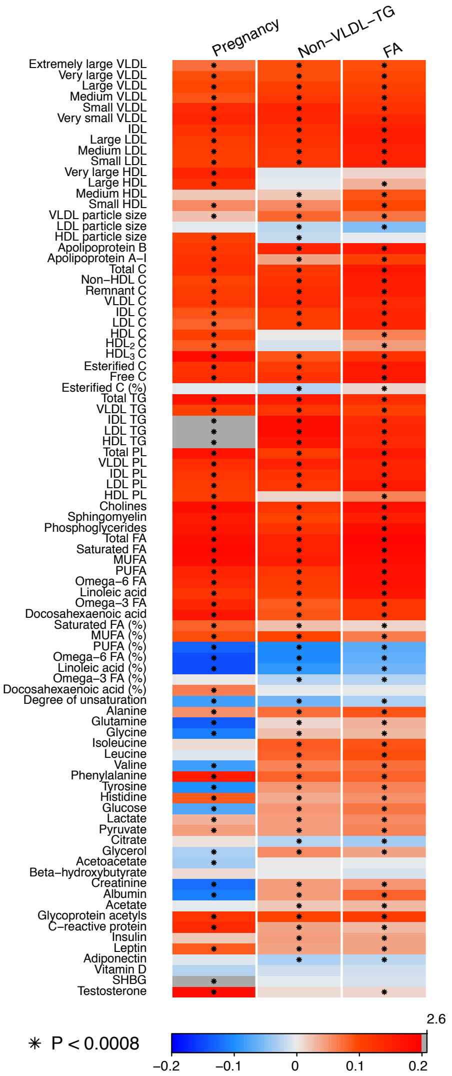

4. A related point - if the metabolite measurements are adjusted for a routinely accessible parameter such as total TAG or LDL-C how many of the associations remain (and with what effect size)? I think this is valuable for the wider audience to understand the added value. If the correlations are too high for such adjustments, a stratified approach as described above could be used.

We agree that within the set of metabolites we have explored in this study many are biologically related and/or epidemiologically correlated and there may be some value in exploring independence. However, we feel presenting results individually for each metabolite is the most meaningful approach as this allows readers to understand relationships across multiple metabolites using knowledge of their biological relationships - in essence in this paper we largely see metabolites that are biologically related to each other and thus also changing together (if that were not the case that would - as the reviewer alludes to - be important and interesting to discuss).

We show the main results adjusted for serum triglycerides and LDL-C in the figures below. In fact, the results illustrate how tricky this type of adjustments are to interpret; it appears that for almost all the lipoprotein and lipid measures the effect of adjusting for serum triglycerides has a stronger effect than for adjusting for LDL-C (though we might expect a more triglyceride/cholesterol-focused behaviours). In this particular case, the explanation is most likely the markedly increased triglycerides due to pregnancy (because triglycerides are the known key nutrient for the developing baby) – however, the biophysically viable (operational) structure of lipoprotein particles always calls for a certain number of cholesterol (both free and esterified) to accompany the transported triglyceride molecules. We think that trying to interpret the effects of these adjustments in more detail than this does not make much sense here. On-the-other-hand, the adjusted results for the low-molecular-weight metabolites indicate that many of these measures are independent of triglycerides and LDL-C. Overall, the extensive metabolic profile, including details and subtleties in relation to lipoprotein metabolism, provides comprehensive understanding of the molecular effects of pregnancy that cannot be captured by routine lipid markers.

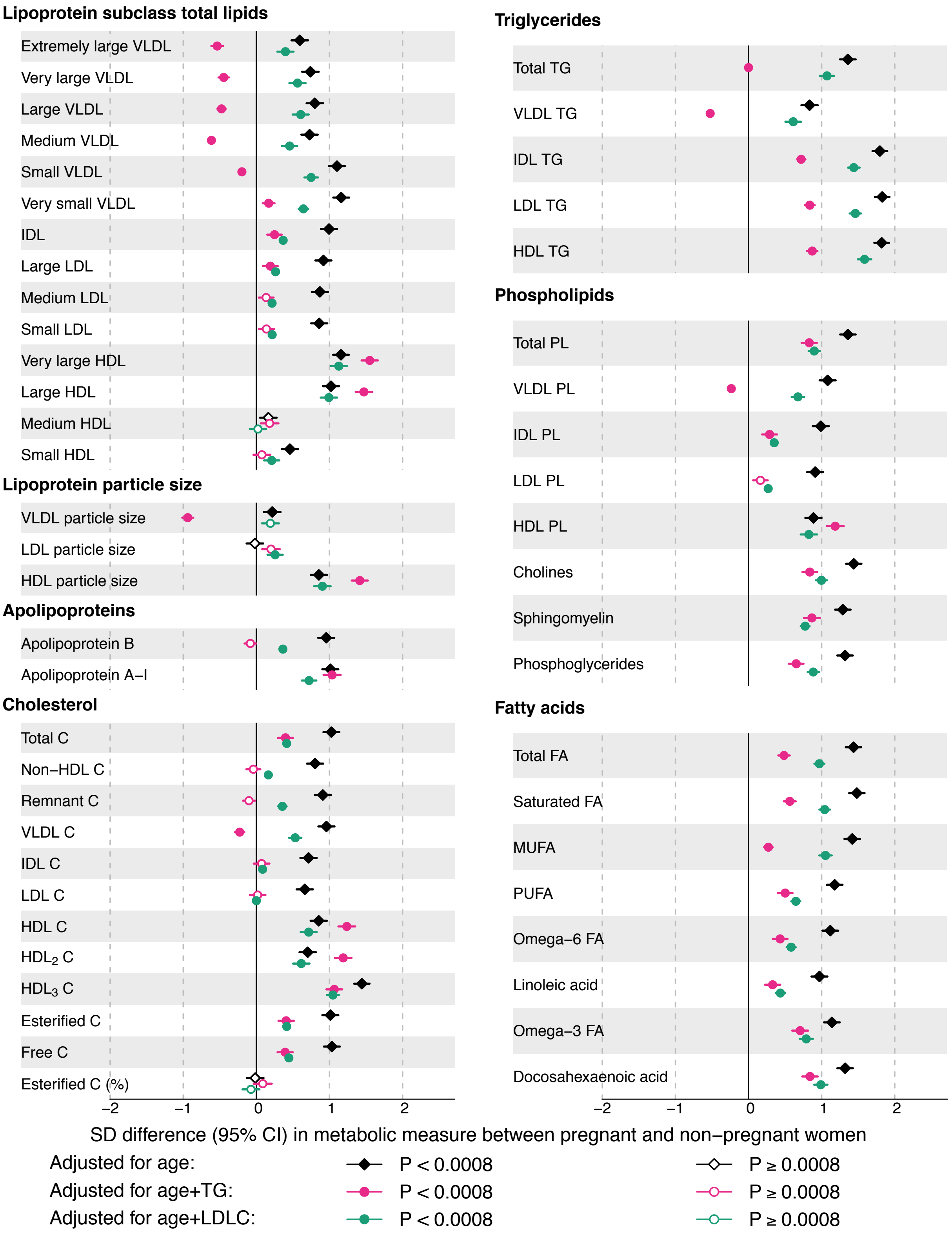
Lipoprotein subclass total lipids
Extremely large VLDL
Very large VLDL
Large VLDL
Medium VLDL
Small VLDL
Very small VLDL
IDL
Large LDL
Medium LDL
Small LDL
Very large HDL
Large HDL
Medium HDL
Small HDL
Lipoprotein particle size
VLDL particle size
LDL particle size
HDL particle size
Apolipoproteins
Apolipoprotein B
Apolipoprotein A–I
Cholesterol
Total C
Non–HDL C
Remnant C
VLDL C
IDL C
LDL C
HDL C
HDL$_2$ C
HDL$_3$ C
Esterified C
Free C
Esterified C (%)
−2
−1
0
1
2
Triglycerides
Total TG
VLDL TG
IDL TG
LDL TG
HDL TG
Phospholipids
Total PL
VLDL PL
IDL PL
LDL PL
HDL PL
Cholines
Sphingomyelin
Phosphoglycerides
Fatty acids
Total FA
Saturated FA
MUFA
PUFA
Omega–6 FA
Linoleic acid
Omega–3 FA
Docosahexaenoic acid
−2
−1
0
1
2
SD difference (95% CI) in metabolic measure between pregnant and non–pregnant women
Adjusted for age:
Adjusted for age+TG:
Adjusted for age+LDLC:
P < 0.0008
P < 0.0008
P < 0.0008
P ≥ 0.0008
P ≥ 0.0008
P ≥ 0.0008

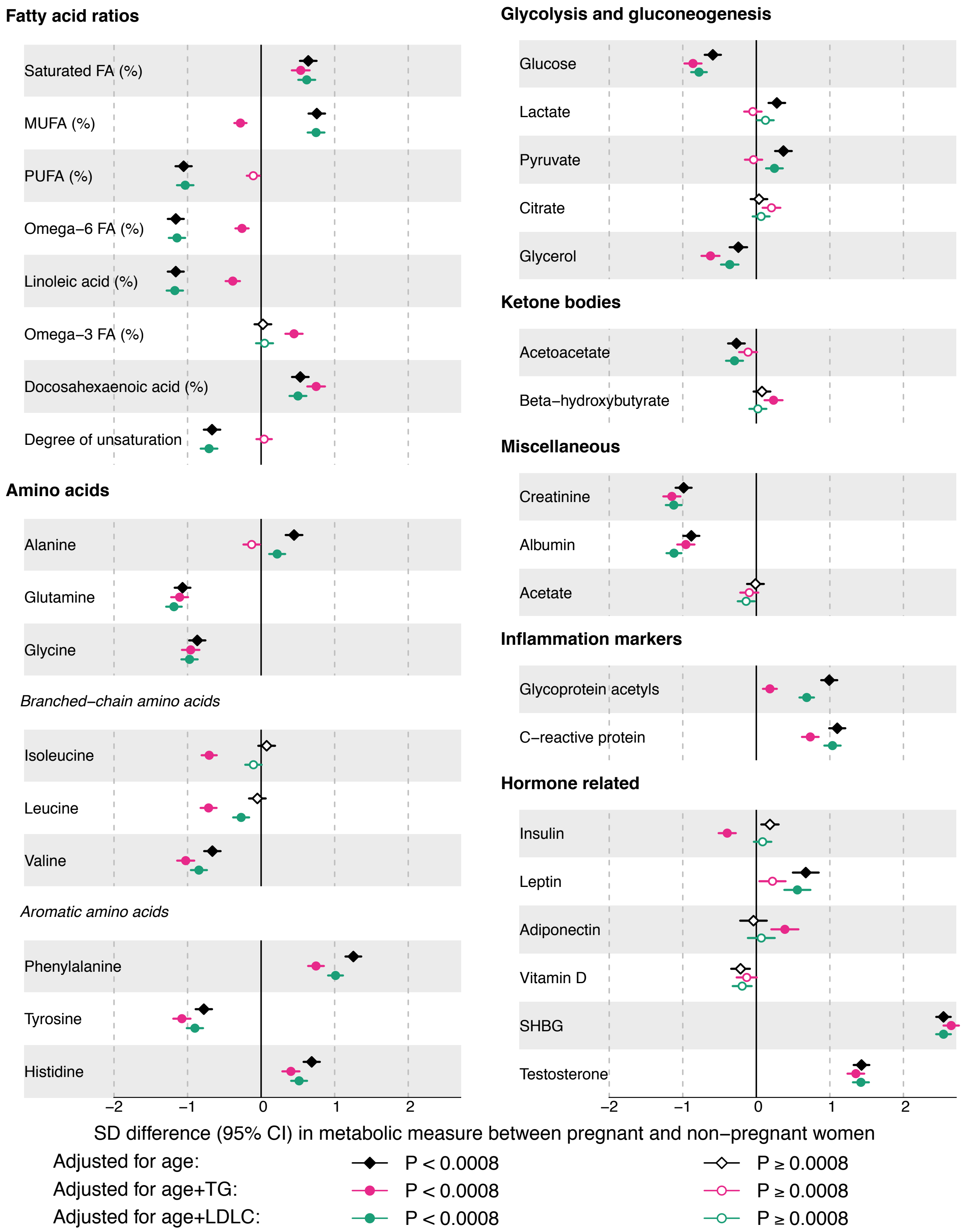
Fatty acid ratios
Saturated FA (%)
MUFA (%)
PUFA (%)
Omega−6 FA (%)
Linoleic acid (%)
Omega−3 FA (%)
Docosahexaenoic acid (%)
Degree of unsaturation
Amino acids
Alanine
Glutamine
Glycine
Branched−chain amino acids
Isoleucine
Leucine
Valine
Aromatic amino acids
Phenylalanine
Tyrosine
Histidine
Glycolysis and gluconeogenesis
Glucose
Lactate
Pyruvate
Citrate
Glycerol
Ketone bodies
Acetoacetate
Beta−hydroxybutyrate
Miscellaneous
Creatinine
Albumin
Acetate
Inflammation markers
Glycoprotein acetyls
C−reactive protein
Hormone related
Insulin
Leptin
Adiponectin
Vitamin D
SHBG
Testosterone
−2
−1
0
1
2
SD difference (95% CI) in metabolic measure between pregnant and non−pregnant women
Adjusted for age:
Adjusted for age+TG:
Adjusted for age+LDLC:
P < 0.0008
P ≥ 0.0008

5. The metabolite with one of the largest discrepancies between the cross-sectional and longitudinal data is DHA (Fig 3, pregnant at follow up but not at baseline, vs CS, or Figure 4 left panel), which the authors rightly indicate is a critical for foetal development. Do the authors have any explanation? Are there any (pre?)analytical concerns with stability of DHA in these samples? This comment could be added to the body text.

We assume that the reviewer is indicating the differences in the point estimates of the ratio measure, the proportion of DHA relative to total fatty acids (DHA%). However, if we look at the larger confidence intervals (that are overlapping) in the longitudinal analyses (due to smaller number of women than in the cross-sectional analysis) there is no apparent discrepancy in the results. The results are clearly consistent for the absolute circulating concentration of DHA. Larger longitudinal studies would be required to ascertain these findings on DHA% in pregnancy.

However, we think these findings are unlikely to be of experimental origin. We have previously published comparisons of fatty acid measures from the NMR platform and gas chromatography; these two technologies show a high level of concordance, in the case of DHA and DHA% the Pearson's correlation coefficients were 0.95 and 0.94, respectively (Würtz et al. Circulation. 2015;131:774-85; Supplemental Figure 12). Further, the quantitative NMR platform applied in this work has a high level of analytical robustness; the CV% for DHA and total fatty acids (for thousands of consecutive samples) were 8.7% and 4.7%, respectively (Kettunen et al. Nat Commun. 2016;7:11122; Supplementary Table 8).

We added a comment on the findings related to DHA and DHA% in the results section (page 14).

Comments from the Editor:

Editorial Requests

Please note that all submissions to BMC Medicine must comply with our editorial policies. Please read the following information and revise your manuscript as necessary. If your manuscript does not adhere to our editorial requirements this will cause a delay whilst the issue is addressed. Failure to adhere to our policies may result in rejection of your manuscript.

Ethics:
If your study involves humans, human data or animals, then your article should contain an ethics statement which includes the name of the committee that approved your study.
If ethics was not required for your study, then this should be clearly stated and a rationale provided.

Consent:

If your article is a prospective study involving human participants then your article should include a statement detailing consent for participation.
If individual clinical data is presented in your article, then you must clarify whether consent for publication of these data was obtained.

We have added an ethics and consent statement in the section ‘Ethics approval and consent to participant’ under the heading ‘Declarations’. The names of the ethics committees that approved the study protocols are now specified.

Availability of supporting data:
BioMed Central strongly encourages all data sets on which the conclusions of the paper rely be either deposited in publicly available repositories (where available and appropriate) or presented in the main papers or additional supporting files, in machine-readable format whenever possible. Authors must include an Availability of Data and Materials section in their article detailing where the data supporting their findings can be found. The Accession Numbers of any nucleic acid sequences, protein sequences or atomic coordinates cited in the manuscript must be provided and include the corresponding database name.

We already had the ‘availability and materials’ section at the submission. We have now added more details into this section.

Authors Contributions:
Your 'Authors Contributions' section must detail the individual contribution for each individual author listed on your manuscript.

We had the ‘Author’s Contribution’ section at the submission. Each individual’s contribution to this manuscript was listed. In the revision phase, we have added one co-author, Dr. Ville-Petteri Mäkinen, who made a key contribution related to the multivariate correlation network analysis requested by the review #3 and now added in the revised version of the manuscript.
